# In Silico-Based Design and In Vivo Evaluation of an Anthranilic Acid Derivative as a Multitarget Drug in a Diet-Induced Metabolic Syndrome Model

**DOI:** 10.3390/ph14090914

**Published:** 2021-09-10

**Authors:** Héctor González-Álvarez, Astrid Bravo-Jiménez, Matilda Martínez-Arellanes, Gabriela Odette Gamboa-Osorio, Edwin Chávez-Gutiérrez, Lino A. González-Hernández, Karina Gallardo-Ignacio, Osvaldo J. Quintana-Romero, Armando Ariza-Castolo, Christian Guerra-Araiza, Laura Martino-Roaro, Dulce María Meneses-Ruiz, Rodolfo Pinto-Almazán, Marco A. Loza-Mejía

**Affiliations:** 1 Design, Isolation, and Synthesis of Bioactive Molecules Research Group, Chemical Sciences School, Universidad La Salle-México, Benjamín Franklin 45, Mexico City 06140, Mexico; hector.gonzalezalvarez@oicr.on.ca (H.G.-Á.); bravojia@myumanitoba.ca (A.B.-J.); matilda.martinez@lasallistas.org.mx (M.M.-A.); gabrielagamboa@lasallistas.org.mx (G.O.G.-O.); lauramartinor@gmail.com (L.M.-R.); 2 Department of Pharmacology and Toxicology, University of Toronto, Toronto, ON M5S 1A8, Canada; 3 Department of Physiology and Pathophysiology, Rady Faculty of Health Sciences, University of Manitoba, Winnipeg, MB R3E 0J9, Canada; 4 Molecular Biology in Metabolic and Neurodegenerative Diseases Laboratory, Research Unit, High Speciality Regional Hospital of Ixtapaluca (HRAEI), Carretera Federal México-Puebla Km 34.5, Ixtapaluca 56530, Mexico; chz_edwin.bioexp@hotmail.com (E.C.-G.); l1994_34@hotmail.com (L.A.G.-H.); karina2107@live.com.mx (K.G.-I.); 5 Department of Chemistry, Center for Research and Advanced Studies of the National Polytechnic Institute (CINVESTAV-IPN), Av. Instituto Politécnico Nacional 2508, Mexico City 07360, Mexico; oquintana@cinvestav.mx (O.J.Q.-R.); aariza@cinvestav.mx (A.A.-C.); 6 Medical Research Unit in Pharmacology, Specialities Hospital Bernardo Sepúlveda, National Medical Center XXI Century, Social Security Mexican Institute (IMSS), Av. Cuauhtémoc 330, Mexico City 06720, Mexico; Christianguerra2001@gmail.com; 7 Incarnate Word University Center, Tlacoquemecatl 433, Mexico City 03100, Mexico; 8 Noncommunicable Diseases Research Group, Universidad La Salle-México, Benjamín Franklin 45, Mexico City 06140, Mexico; dulce.meneses@lasalle.mx

**Keywords:** metabolic syndrome, multitarget drug, molecular docking, in vivo evaluation, toxicity

## Abstract

Metabolic syndrome (MetS) is a complex disease that affects almost a quarter of the world’s adult population. In MetS, diabetes, obesity, hyperglycemia, high cholesterol, and high blood pressure are the most common disorders. Polypharmacy is the most used strategy for managing conditions related to MetS, but it has drawbacks such as low medication adherence. Multitarget ligands have been proposed as an interesting approach to developing drugs to treat complex diseases. However, suitable preclinical models that allow their evaluation in a context closer to a clinical situation of a complex disease are needed. From molecular docking studies, compound **1b**, a 5-aminoanthranilic acid derivative substituted with 4′-trifluoromethylbenzylamino and 3′,4′-dimethoxybenzamide moieties, was identified as a potential multitarget drug, as it showed high in silico affinity against targets related to MetS, including PPAR-α, PPAR-γ, and HMG-CoA reductase. It was evaluated in a diet-induced MetS rat model and simultaneously lowered blood pressure, glucose, total cholesterol, and triglyceride levels after a 14-day treatment. No toxicity events were observed during an acute lethal dose evaluation test at 1500 mg/kg. Hence, the diet-induced MetS model is suitable for evaluating treatments for MetS, and compound **1b** is an attractive starting point for developing multitarget drugs.

## 1. Introduction

Metabolic syndrome (MetS) is a complex disease that has become one of the major public health challenges worldwide. It is a clustering of metabolic abnormalities such as abdominal obesity, hyperglycemia, atherogenic dyslipidemia, and hypertension [1]. According to several organizations like the World Health Organization (WHO), the Adult Treatment Panel III (ATP III), and the International Diabetes Federation (IDF), MetS can be diagnosed through the study of these disorders [2,3]. The exact mechanisms implicated in the pathogenesis of MetS are still not fully understood. Among the factors that increase the probability of developing MetS are genetic background, diet, physical activity, age, gender, over- and undernutrition, and body habits. The combination of these factors can lead to the development of visceral obesity, insulin resistance, adipokine dysregulation, and low-grade chronic inflammation, which appear to be the major players in the progression of MetS [4]. It is estimated that approximately 20–25% of the world’s adult population suffer MetS. They are twice as likely to die from some of their complications and three times as likely to have a stroke or heart attack than people without the syndrome [3].

MetS treatment must begin with lifestyle changes [5]. Modifications in diet quality, calorie intake limitation, physical activity increment, and medical follow-up are the cornerstones of this strategy [6]. However, when pharmacological intervention is needed, each component of MetS is typically treated separately [1,7]. Polypharmacy, which could be defined as the administration of multiple drugs that will hit multiple targets simultaneously [8], is the current strategy employed for the pharmacological management of MetS. However, polypharmacy can lead to ineffectiveness and low medication adherence due to drug-drug interactions, which may cause variations in the concentration of one or more of the drugs, increasing the probability of adverse effects [9]. Hence, multitarget drugs are attractive for treating complex diseases like MetS [10,11,12,13].

**Figure 1:**
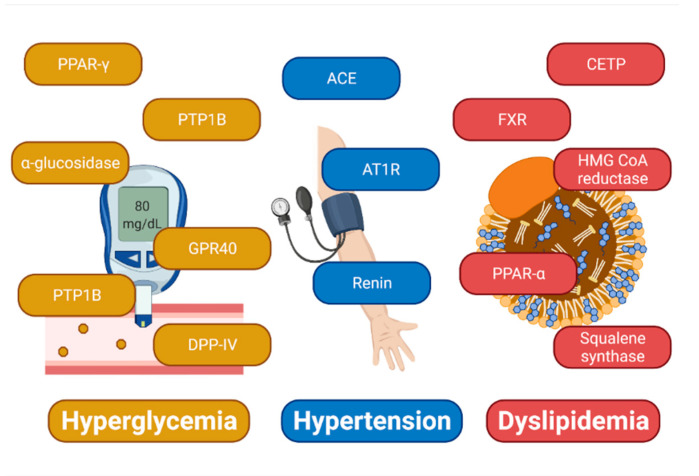
Examples of drug targets proposed for the treatment of components of MetS.

The development of multitarget drugs has become an attractive field of research for medicinal chemists and pharmacologists [14,15]. In fact, the number of multitarget drugs approved by the Food and Drug Administration has been increasing since 2015 [16,17]. One of the strategies for designing multitarget ligands is to integrate two or more pharmacophoric fragments in one molecule [18]. Therefore, the design of a multitarget drug for a complex disease should begin with selecting appropriate targets, using some strategies like high-throughput screening or network pharmacology [19], although this is not always an easy task [20]. Several studies have found that the dysregulation in the mechanisms of some enzymes and receptors is related to the physiopathology and complications of MetS. Some drug targets have been proposed from these studies and have been used to develop clinically used drugs (Figure 1) [12,21,22,23].

After deciding on the drug targets, an initial molecular scaffold should be selected for proposing modifications that may lead to a potential hit. The selection of this initial template could be carried out using a pharmacophore and docking-based strategy, which intends to find a common pharmacophore to the selected targets or a de novo-based design method that aims to construct a tailored ligand from an initial structure or fragments [24]. Among privileged scaffolds used in medicinal chemistry, anthranilic acid exhibits many biological activities, including antibiotics, antivirals, anti-inflammatories, and antitumorals [25,26,27,28,29,30]. Moreover, some anthranilamide derivatives have shown modulating activity on peroxisome proliferator-activated receptors (PPAR) and the farnesoid X receptor (FXR), which are involved in mechanisms of metabolic balance [31,32,33]. Some common structural features that the compounds of these studies share are the incorporation of aromatic rings bearing hydrogen-bonding functional groups in the carboxylic group of position 1 and voluminous substituents bound to the amino group in position 2 of the anthranilic acid core. Therefore, anthranilic acid is a suitable molecular template for the design of multitarget drugs for metabolic diseases.

Biological evaluation of a multitarget drug is not an easy task. While valuable information is provided from in vitro assays or in vivo experiments on one specific component of a complex disease, suitable in vivo models that allow fast screenings and give relevant information that allows quick go or no-go decisions are also plausible [34,35,36]. In our case, an appropriate in vivo model should simultaneously replicate the components of MetS in humans, such as obesity, dyslipidemia, hypertension, and diabetes. In genetically modified animals, the susceptibility to the different components of MetS may be higher. However, these models present some disadvantages: some display genetic mutations rarely observed in humans. Therefore, they do not represent most of the population, and their reproduction and maintenance require a significant economic investment [37]. Diet is a factor that can influence the development of MetS by inducing metabolic imbalances in humans and animals. Previous reports have correlated the increase in MetS with the high intake of saturated fats and carbohydrates such as fructose and sucrose, known as the Western diet [38,39]. In animal models, diets with less than 10% of calories derived from fats, high in fat (saturated fats), or high carbohydrate content (fructose or sucrose) have been reported to increase glucose triacylglycerides and cholesterol plasma levels constituting diet-induced MetS models [38,40,41].

In this work, we present the docking-based design of anthranilic acid derivatives as potential multitarget ligands for the management of MetS, the synthesis of the selected candidate, its in vivo evaluation in a diet-induced obesity model, and acute lethal dose assessment through Lorke’s method.

## 2. Results

### 2.1. Docking-Based Design

**Figure 2:**
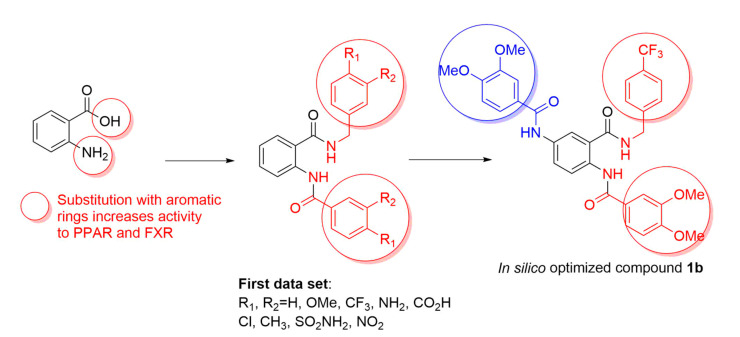
Docking based design of anthranilamides as multitarget drugs.

Centered on anthranilic acid, we proposed a first data set composed of 90 molecules (the list of ligands **1a–90a** is described in Supplementary Table S1) based on benzyl substituted derivatives (Figure 2) and calculated their theoretical affinity against the drug targets shown in Figure 1 using molecular docking. The targets were assorted according to their association to one of the factors related to MetS, as seen in Figure 1. Then, the most promising ligands were selected based on their theoretical affinity to targets of two or more groups.

The ligands of this first data set displayed higher or similar theoretical affinity compared to those of known ligands of PPAR-α, PPAR-γ, angiotensin-converting enzyme (ACE), and HMG-CoA reductase, as shown in Table 1. Low affinity was predicted for CEPT, squalene synthase, renin, DDP-IV, and GPR40. A complete table is included as part of supplementary information. From this data, we concluded that the presence of 4′-trifluoromethyl substitution in the benzylamine ring and 3′,4′–methoxy disubstitution in the 2–benzamide moiety increased theoretical affinity to these particular targets.

**Figure 3:**
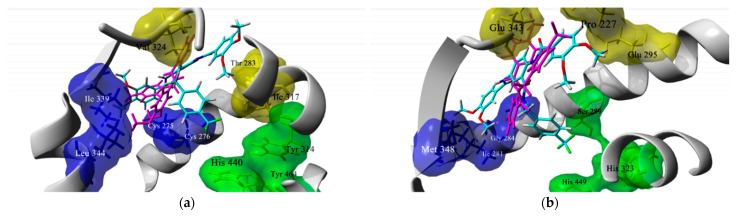
Predicted docking poses for compound **48a** (magenta) and **1b** (CPK, carbon atoms in cyan) in their complexes with (**a**) PPAR-α; (**b**) PPAR-γ. Green and blue-colored residues correspond to the binding pockets of known dual PPAR partial agonists, and yellow residues describe additional binding sites. See discussion section for details.

**Figure 4:**
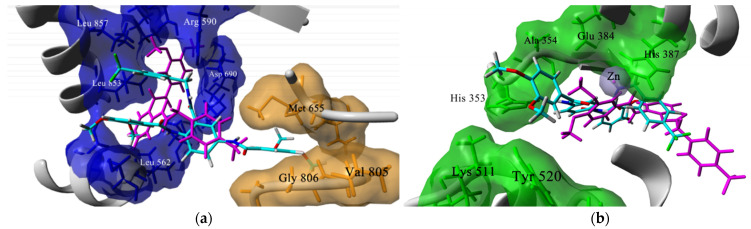
Predicted docking poses for compound **48a** (magenta) and **1b** (CPK, carbon atoms in cyan) in their complexes with (**a**) HMG-CoA reductase; (**b**) angiotensin-converting enzyme (ACE). In Figure 4a, blue- and orange-colored residues correspond to the HMG-CoA and NADP-binding sites, respectively.

On the other hand, the analysis of the predicted poses (Figure 3 and Figure 4) revealed additional pockets that could be used to add a substituent in position 5 of the anthranilic acid template, particularly in PPAR-α and PPAR-γ predicted poses. This observation led to the design of 5-benzamide substituted derivatives which exhibited even higher theoretical affinities to PPAR-α, PPAR-γ, ACE, and HMG-CoA reductase, particularly compound **1b,** as shown in Table 1. Moreover, this ligand possesses an acceptable in silico ADME/Tox profile predicted using pkCSM [42], including high intestinal absorption (75%), poor permeability to CNS, and negative results on Ames test, hepatotoxicity, skin sensitization, and hERG I inhibition. The results of these in silico studies and previous studies of anthranilamide derivatives [30,31,32] prompted the selection of this compound for evaluation in the in vivo diet-induced MetS model.

### 2.2. Preparation of Compound ***1b***

**Scheme 1:**
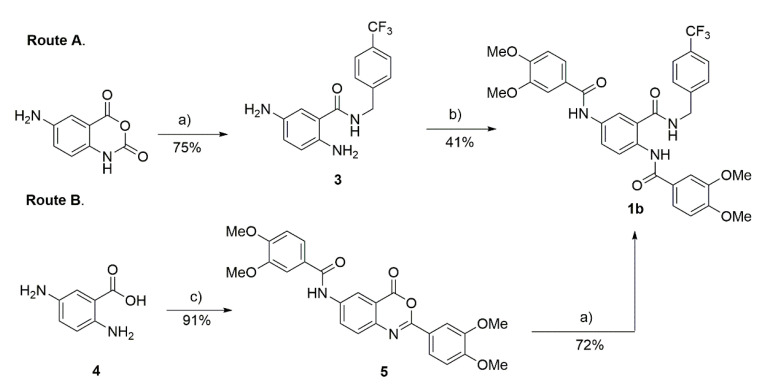
Synthetic routes carried out for the preparation of compound **1b**. (a) 4-trifluoromethylbenzylamine, DMF, room temperature; (b) 2 equivalents of 3,4-dimethoxybenzoyl chloride, pyridine, 0 °C; (c) 3 equivalents of 3,4-dimethoxybenzoyl chloride, pyridine, 0 °C.

The synthetic strategies followed for the preparation of compound **1b** are outlined in Scheme 1. Route B presented higher yields and more straightforward purification procedures, which are shown in the methodology section. Compound **1b** was characterized through IR, ^1^H NMR, ^13^C NMR, MS, and elemental analysis data.

### 2.3. In Vivo Evaluation

**Figure 5:**
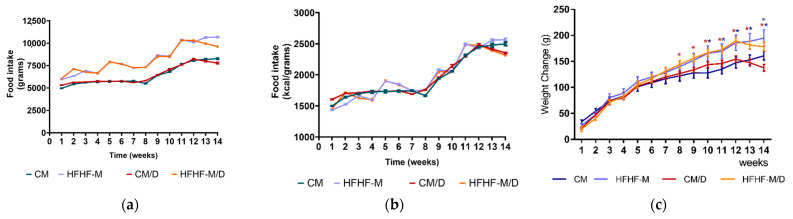
Effect after 14 days of compound **1b** administration on food intake and weight change on the MetS animal model (**a**) food consumption, (**b**) calorie intake, (**c**) weight change. HFHF-M and HFHF-M/D groups presented a statistically significant increase in kg/cal consumption compared to control (CM and CM/D) groups, observing significant weight differences between groups from week 8 to week 12. After 14 days of treatment with compound **1b**, the HFHF-M/D group had a reduction of weight change on both treated groups (CM/D and HFHF/D) being non-statistically significant between HFHF-M and HFHF-M/D. Data are presented as means ± standard deviation (SD). * Represents statistically significant differences between groups compared to their own control group (CM vs. HFHF-M) (CM/D vs. HFHF-M/D) (*p* < 0.05). The color of the * represents which group is the compared to their control.

For in vivo evaluation, we used a diet-induced MetS model [41]. In this model, MetS is induced through 12 weeks of high fructose-high fat (HFHF) diet. First, the animals were assigned to a group with a standard diet or to the HFHF diet group. We observed that, during the phase of MetS induction, food consumption was similar in both groups, while calorie intake was significantly higher in the HFHF group, as seen in Figure 5. After the induction phase, the animals of the standard diet were randomly assigned to two groups, one (CM/D group) would receive compound **1b** for 14 days (10 mg/kg, p.o.), and the other group would receive no treatment (CM group). The same assignment was performed for the HFHF group (HFHF-M received no treatment, while HFHF-M/D received compound **1b**, 10 mg/kg, p.o for 14 days). In the groups treated with compound **1b**, a significant decrease in food consumption and calorie intake was observed compared to the control groups (Figure 5a,b), resulting in a reduction in body weight. However, this reduction was not significant between the HFHF-M and HFHF-M/ D groups (Figure 5c).

**Figure 6:**
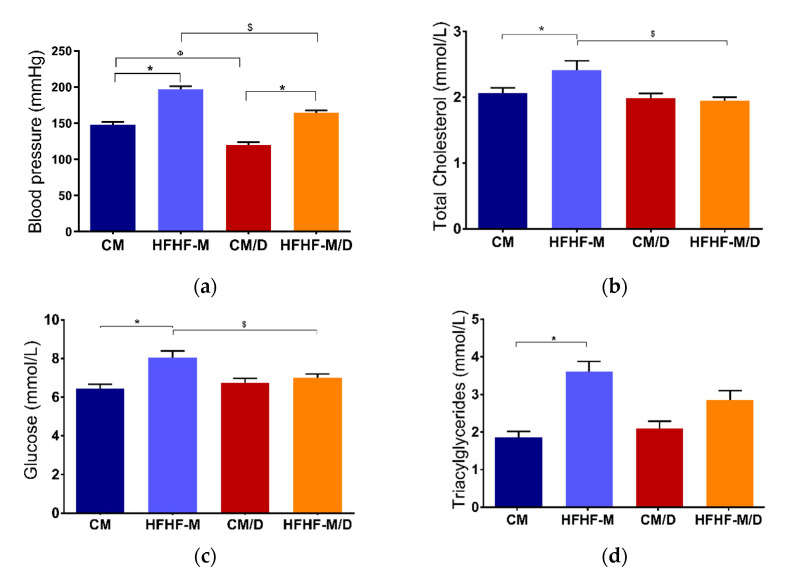
Effect after 14 days of compound **1b** administration on metabolic parameters associated to MetS on the in vivo model (**a**) Blood Pressure (**b**) Cholesterol, (**c**) Glucose, (**d**) Triacylglycerides. After 14 days of treatment with compound **1b**, the HFHF-M/D group had a statistically significant reduction of blood pressure, total cholesterol, and glucose compared to the HFHF-M group. Also, the HFHF-M/D group observed a non-statistically reduction of the triacylglycerides compared to the HFHF-M group, but also a non-statistical significant difference was observed to CM and CM/D groups. Data are presented as means ± standard deviation (SD). * Represents statistically significant differences between groups compared to their own control group (CM vs. HFHF-M) (CM/D vs. HFHF-M/D) (*p* < 0.05). Φ Represents statistically significant differences between CM vs. CM/D (*p* < 0.05). $ Represents statistically significant differences between HFHF-M vs. HFHF-M/D (*p* < 0.05).

Regarding the effects of the 14-day treatment with compound **1b** on blood pressure, total cholesterol plasmatic concentration, and glycemia levels, a statistically significant decrease was observed in the groups treated with compound **1b** compared with the non-treated groups (*p* < 0.05), as shown in Figure 6. However, no significant decrease was observed in triglyceride plasma levels (*p* = 0.06). A table with mean values and the standard deviation is presented in Table S2.

Finally, acute in vivo toxicity was evaluated by oral administration using an adaptation of the Lorke method [36], reaching a maximum dose of 2500 mg/kg of body weight, without any acute toxicity event being recorded at the end of the study. Only at the dose of 2500 mg/kg did one of the study animals present behavioral alterations that suggested some effects in the CNS, but only at very high doses.

## 3. Discussion

### 3.1. In Silico Studies

Drugs can be discovered by several strategies. Commonly, in vitro experiments are used to measure the activity of a library of compounds against a given target. However, especially in multitarget drug discovery, this process could be expensive and time-consuming. Therefore, we decided to perform an in silico + in vivo screening approach that could help us identify promising multitarget drug candidates in a holistic diet-induced model of MetS, faster and more affordably. Using such holistic models that represent the complexity and the pathophysiological characteristics of the MetS or other complex diseases is an attractive option for evaluating multitarget drugs instead of evaluating the most promising molecule in several models that represent only one factor at a time. Using the diet-induced MetS model, we have identified compound **1b** as a potential multitarget ligand, as it has exhibited multiple effects on metabolic parameters related to the components of MetS.

After determining the docking scores of the anthranilic acid derivatives library against some targets related to MetS, we observed that compound **48a** presented balance in theoretical affinities. Outstandingly, we noticed that the theoretical affinities of this molecule against PPAR-α, PPAR-γ, HMG-CoA reductase, ACE, and aldose reductase were close to those of the reference ligands. Hence, this could mean that it could possess multitarget properties that could give this molecule an interesting therapeutic profile in the management of MetS. Partial agonists of PPAR-α (like fibrates) and PPAR-γ (like glitazones) are clinically used for the management of dyslipidemia and hyperglycemia, respectively. HMG-CoA inhibitors (like statins) are used for decreasing LDL cholesterol levels. ACE inhibitors are well-known antihypertensives, and aldose reductase inhibitors have been tested as drugs for diminishing the effects of diabetic neuropathy [4,43]. The multitarget effects of some clinically used drugs employed to treat one component of MetS and their potential benefits have been described in the literature, i.e., the anti-inflammatory effect of statins [44] or the PPAR-γ agonistic effect of telmisartan, an angiotensin-II receptor blocker [45]. Indeed, a dual-target or a multitarget drug has its drawbacks because it should not be used in patients with only one dysregulated metabolic parameter. However, since most patients with MetS have at least two risk factors [16,46], multitarget drugs must be taken into consideration among the potential therapeutical options.

The analysis of the predicted poses for compound **48a** on the mentioned targets revealed the possibility of including an additional third ring attached to anthranilic acid to induce further interactions, as seen in Figure 3 and Figure 4. This information led to the design of new trisubstituted compounds that possess trifluoromethyl and 3,4-dimethoxy substitution patterns in the benzylamine and benzoyl moieties in a similar fashion as compound **48a**. From these studies, we found that compound **1b** was the most promising analog since it presents lower Rerank Score values (meaning a greater binding affinity) for practically all the targets than the other trisubstituted analogs we designed. The predicted poses for compound **1b** in PPAR-α, PPAR-γ, HMG CoA reductase, and ACE, also shown in Figure 3 and Figure 4, revealed that the incorporation of the additional benzamide moiety had a positive effect on the predicted affinity. Additionally, compound **1b** had a higher theoretical affinity than the cocrystallized ligands and some clinically used drugs used as controls during docking studies.

The binding sites of both PPAR-α and PPAR-γ have been described as a Y-shaped pocket [47,48]. One of the arms of this Y-shaped pocket is known as the acid-binding site, colored in green in Figure 3. Partial agonism of PPARs do not require direct interaction of ligands with helix 12 (H12) but stabilizing other regions of the binding pocket, such as H3 and which the interaction with the residues of this pocket plays an important role in the binding of several selective and dual PPAR agonists [49,50]. As seen in Figure 3, incorporating an additional ring to **48a** to render **1b** allows the interaction of the trifluoromethyl substituted ring with the acid-binding site, particularly on PPAR-γ. On the other hand, fibrates and thiazolidinediones, which are well-known ligands of PPAR-α and PPAR-γ, respectively, adopt a more linear conformation within the binding site occupying only two of the three arms of the binding site, which are colored in green and blue in Figure 3. It has been recently proposed that ligands capable of occupying the three arms of the Y-shaped pocket could display higher potency and better safety profile like pemafibrate [47,51]. As seen in Figure 3, compound **1b** can occupy the three arms of the binding pocket (colored in yellow). Therefore, it could be a dual PPAR α/γ ligand with good potency. Figure 4 depicts the predicted binding modes of compounds **48a** and **1b** to HMG-CoA reductase and ACE. In both enzymes, compound **48a** occupies the same binding sites as reference compounds atorvastatin and lisinopril, which are competitive inhibitors. However, compound **1b** could act as a mixed inhibitor in HMG-CoA reductase as it could also bind to the NADP site.

A complete prediction of pharmacological relevant properties must include the in-silico evaluation of the pharmacokinetic and toxicological (ADME /Tox) profiles. We used pkCSM, which uses graph-based signatures to predict ADME/Tox properties [42]. The most relevant features, included predicting good oral absorption and low toxicity and carcinogenicity, desirable qualities for any candidate molecule. Based on molecular docking results that suggested multitarget properties of compound **1b** and the favorable predicted ADME/Tox profile, we decided to evaluate this molecule in a holistic model of MetS that simultaneously reproduces its characteristic features.

### 3.2. Preparation of Compound ***1b***

We proposed two strategies for the synthesis of 1b: The initial strategy (route A, Scheme 1) began from 5-aminoisatoic anhydride treated with 4-trifluoromethylbenzylamine. The resulting benzylamide product (**3**) was treated with two equivalents of 3,4-dimethoxybenzoyl chloride to give compound **1b** in 41% yield after a complex purification procedure. The main subproduct formed was identified as a quinazolinone derivative which could be expected as this is a reported route for preparing these heterocycles [52]. Thus, a different strategy was proposed (route B, Scheme 1). We began from 2,5-diaminobenzoic acid **4**, which was treated with three equivalents of 3,4-dimethoxybenzoyl chloride to give the benzoxazinone **5** derivatives in 91% yield [53,54]. Afterward, **5** was treated with 4-trifluoromethylbenzylamine at room temperature for 24 h to give **1b** in 72% yield. In this case, a more straightforward purification method was used.

Compound **1b** was isolated as a white solid. As we wanted to administrate it orally in the in vivo experiments, we prepared a formulation of **1b** using polyoxyl-35 castor oil (Kolliphor EL^®^, BASF (Ludwigshafen, Germany)) at 2% in water as previously reported [55], rendering a stable white emulsion.

### 3.3. In Vivo Evaluation

Since PPARs regulate the expression of numerous genes involved in the metabolism of fatty acids, lipoproteins, blood pressure, and glycemic control [56,57], and considering the theoretical affinity of compound **1b** on the PPAR-α and PPAR-γ receptors and some other targets related to MetS; we expected that compound 1b would exhibit multitarget properties. Hence, the effect of 1b on the parameters associated with MetS was evaluated in a standardized animal model, where MetS was induced by a diet high in fructose and fat to observe if its administration could have some simultaneous effect on glucose, cholesterol, and triacylglyceride levels and blood pressure.

We observed that the administration of compound **1b** in Sprague-Dawley adult rats significantly reduced food consumption and body weight gain over the 14-day treatment period (Figure 5). In agreement, Larsen et al. reported a reduction in food intake and body weight in animals fed a high-fat diet and in the control group treated with 3 mg/kg/day of ragaglitazar (a dual agonist of PPAR-α/γ). Nevertheless, the decrease in feed intake and body weight in the animals treated with ragaglitazar was smaller than the effects observed in the fenofibrate (PPAR-α agonist) treated animals [58]. Therefore, the effect observed with compound **1b** treatment could be similar to ragaglitazar, both being PPAR-α/γ dual agonists. Likewise, Wang et al. reported a decrease in food consumption and gain in body weight in obese Zucker rats after the administration for 14 days with nitro-oleic acid (OA-NO_2,_ an endogenous agonist of PPAR) [59]. Due to the pharmacological characteristics predicted for **1b**, it could regulate the activity of PPAR-α as an agonist in the metabolism of lipids and affect satiety [60,61].

On the other hand, compound **1b** administration significantly reduced blood pressure which could be explained through ACE inhibition since it was predicted that it could be a potential target of **1b**. Such inhibition may attenuate angiotensin-I’s conversion into angiotensin-II and result in decreased blood pressure and regulation of fluid volume [62]. However, various pleiotropic properties have been reported in PPAR-γ agonists, as well as their metabolic action, including blood pressure [63,64,65]

Concerning total cholesterol levels, a statistically significant decrease in total cholesterol was observed in the HFHF-M/D group compared to the HFHF-M group (Figure 6). Accordingly, de la Heras et al. reported that treatment with rosuvastatin (HMG-CoA inhibitor) reduced serum concentration of triglycerides, very low-density cholesterol, and glucose and insulin in male Wistar rats fed with a high-fat diet. The authors concluded that lowering blood lipids improved insulin sensitivity, thus reducing blood glucose levels [66]. The results in the in silico and in vivo studies obtained in the present study allow us to hypothesize that compound 1b can regulate the biosynthesis of cholesterol by modifying the enzyme HMG-CoA reductase HMG-CoA into mevalonic acid. Such pharmacological effects are observed in hypocholesterolemic drugs such as statins.

The administration of **1b** caused a decrease in triacylglyceride levels, but it was not statistically significant (*p* = 0.06). Several reports like those of Wang et al. and by Shiomi et al. demonstrate that the administration of PPAR agonists in animal models of MetS statistically significantly decreased the serum concentration of triacylglycerides [59,67]. However, the present study did not show any differences between HFHF-M/D groups with the control groups (CM/D and CM). These data suggest that as there is a decrease in serum triglycerides, and it may only be a matter of longer administration time to observe a statistically significant change.

One crucial aspect during preclinical development is to evaluate the potential toxic effect of a drug candidate. Therefore, we considered it important to conduct an acute toxicity test using Lorke’s method [68]. This method considers two phases. In phase one, nine animals were divided into three groups of three animals each and received 10 mg/kg, 100 mg/kg, and 1000 mg/kg, respectively. In the second phase, six animals were assigned to two groups and received 1500 mg/kg and 2500 mg/kg per group. No signs of toxicity and no fatalities were recorded during the 24 h after compound **1b** administration. Only signs of CNS alterations were observed in one animal at the highest dose studied (2500 mg/Kg), giving some evidence of the security of this molecule.

Multitarget drugs have become of interest in the clinical field since they could have a significant impact in multifactorial diseases, where the single-target approach has led to low therapeutic efficacy due to inadequate treatment adherence, drug-drug interactions, or high medication costs. The development of multitarget drugs needs the confirmation of the mode of action through appropriate evaluations, including in vitro testing. However, using holistic models that represent the complexity of a multifactorial disease might be helpful since the findings of a drug evaluation in these models can enlighten the choice of adequate in vitro assays that confirm the molecular mechanisms of the tested molecules.

## 4. Materials and Methods

### 4.1. In Silico Studies

#### 4.1.1. Ligand Design

Taking anthranilic acid as the initial core, we designed a library of 90 *N*-benzylanthranilamides. Several moieties were inserted at positions 3 and 4 of the benzyl of benzyl and benzoyl rings, as shown in Figure 2. The molecular structures were built using Spartan ‘10 for Windows [69] (Wavefunction, Inc., Irvine, CA, USA). Standard fragments and their geometries were optimized using the MMFF force field. After that, these structures were exported to Molegro Virtual Docker 6.0.1(Qiagen Bioinformatics, Aarhus, Denmark) [70]. Additionally, we designed a new series of trisubstituted derivatives. Their structures were also built in Spartan ‘10 and optimized with the same procedure as disubstituted derivatives.

#### 4.1.2. Docking Studies

Molecular docking studies of disubstituted and trisubstituted derivatives were carried out using Molegro Virtual Docker based on the crystal structures retrieved from Protein Data Bank [71] of some targets related to MetS: acyl-coenzyme A:cholesterol acyltransferase (ACAT, PDB ID: 6L47 [72]), aldose reductase (PDB ID: 1US0 [73]), dipeptidyl peptidase 4 (DPP4, PDB ID: 4A5S [74]), angiotensin-converting enzyme (ACE, PDB ID: 1O86 [75]), farnesoid X receptor (FXR, PDB ID: 1OSH [76]), GPR40 (also known as free fatty acid receptor 1 FFAR-1 PDB ID: 4PHU [77]), HMG CoA reductase (PDB ID: 1HWK [78]), PPAR-α (PDB ID: 1I7G [79]), PPAR-γ (PDB ID: 1I7I [79]), protein tyrosine phosphatase 1B (PTP 1B, PDB ID: 1XBO [80]). The standard procedure suggested by the fabricant was used with some modifications as previously reported [81]. Briefly, all the solvent molecules and cocrystallized ligands were removed from the structures. Active sites of each enzyme or the ligand binding domain of the nuclear receptors were chosen as the searching sites and delimited with a 15 Å radius sphere centered on the cocrystallized ligand. The root mean square deviation (RMSD) limit for multiple clusters was set to <1.00 Å. The docking algorithm (MolDock Optimizer) was set to 5000 maximum iterations with a simplex evolution population size of 5000 and 50 runs for each ligand. After docking, the Rerank Score was calculated as the theoretical binding affinity. More negative values are associated with better binding. To test the efficacy of this procedure, the cocrystallized ligands were also docked to their respective receptors, and the RMSD of the lower Rerank score pose from the corresponding crystal coordinates was recorded. In all the docking procedures, the RMSD was lower than 2.0 Å. The in silico ADME/Tox profile was predicted for compound **1b** using the pkCSM tool (http://biosig.unimelb.edu.au/pkcsm/, accessed on 10 January 2021) [42].

### 4.2. Chemistry

All initial reactants and solvents were purchased from Sigma-Aldrich (St. Louis, MO, USA) and used without further purification. Reactions were monitored by TLC on silica gel 60 on aluminum foil from Sigma-Aldrich (St. Louis, MO, USA). IR spectra were obtained in a Perkin Elmer FTIR-670 Plus spectrophotometer in a KBr disk. NMR spectra were recorded using a JEOL ECA-500 JEOL spectrometer (B0 = 11.75 T). The unified scale [82] was used as a primary reference based on the 1H NMR resonance of TMS in a dilute solution (volume fraction *j* < 1%) in chloroform, (*d* 1H, *d* 13C = 0), neat MeNO_2_ (*d* 15N for *X* 15N = 10.136767 %) and CCl_3_F (*d* 19F for *X* 19F = 94.094011 %). The spectrometer is equipped with a 5-mm multinuclear and pulse field gradient probe. The samples were determined in DMSO-*d_6_* solution. ^1^H NMR spectra were recorded at 500.1599 MHz (spectra width 22 ppm, acquisition time 2.8 s, pulse width 45°, 16 scans, and recycle delay of 1 s). ^13^C NMR spectra were recorded at 125.7653 MHz (spectral with 250 ppm, acquisition time 1.04 s, equivalent 30 pulse duration, scans 8000, and recycle delay of 0.1 s). ^15^N NMR spectra were recorded at 50.70 MHz (spectral with 250 ppm, acquisition time 1.3 s, scans 7500). ^19^F NMR spectra were recorded at 470.62 MHz (spectral with 225 ppm, acquisition time 3.05 s, equivalent 30 pulse duration, scans 16, and recycle delay of 2 s). ^15^N NMR spectra determined by INEPT methods. The structures of the compounds were assigned using the COSY-45, pfg-HMBC, and pfg-HSQC pulse sequences [83]. The FAB-MS analyses were obtained on a JEOL Sx102 mass spectrometer.

#### 4.2.1. 2,5-Diaminobenzoic Acid (**4**)

In a round-bottom flask, technical grade sodium dithionite (70%, 30 g, 120 mmol) was added to 200 mL of a 15% sodium hydroxide solution and heated to 80 °C. Then, 4 g of 2-amino-5-nitrobenzoic acid **2** (22 mmol) were slowly added in small portions. Subsequently, the reaction mixture was stirred for one hour. During the reaction time, the orange-color solution gradually turned yellow. After reaction completion, the crude was treated with acetic acid dropwise until it reached pH 7, leading to the formation of a grey precipitate, which was then filtered, washed with cold acetone, and dried under reduced pressure to afford the desired product as a white solid in a 73% yield. m.p. 223–225 °C (dec.); IR (KBr, cm^−1^): 3328 (N–H), 2925 (C–H), 1627 (C=O), 1542 (C=C).

#### 4.2.2. *N*-(2-(3,4-Dimethoxyphenyl)-4-oxo-4H-benzo[d][1,3]oxazin-6-yl)-3,4-DIMETHOXYBENZAMIDE (**5**)

**Figure 7:**
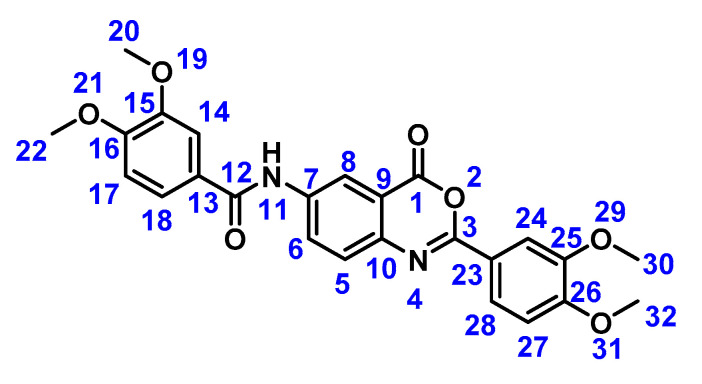
Structure of compound **5** and atom numbering used for NMR characterization.

To a round bottom flask equipped with a magnetic stirrer and ice bath, 100 mL of pyridine were added. Afterward, 3,4-dimethoxybenzoyl chloride (5.24 g, 26.0 mmol) was added and vigorously stirred until its complete dissolution. After 10 minutes, compound **4** (1.3 g, 8.7 mmol) was added and stirred for 2 h at 5 °C. Once the reaction was completed, 250 mL of water were added, and a white precipitate was immediately formed, which was then filtered, washed with ethanol, and dried under reduced pressure to afford the desired product **5** as a yellowish solid in 91% yield. m.*p*. > 250 °C, IR (KBr, cm^−1^): 3335 (N-H), 1709 (C=O, benzoxazinone), 1632 (C=O, amide), 1590 (C=C, aromatic), 1201 (O–CH_3_). ^1^H NMR (DMSO-*d*_6_, 500 MHz) *δ* = 10.44 (1H, s, H11), 8.60 (1H, d, *J* = 2.7 Hz, H8), 8.24 (1H, dd, *J* = 2.7, 9.1 Hz, H6), 7.75 (1H, dd, *J* = 1.8, 8.7 Hz, H28), 7.68 (1H, d, *J* = 8.8 Hz, H5), 7.64 (1H, d, *J* = 1.8 Hz, H24), 7.62 (1H, dd, *J* = 2.1, 8.4 Hz, H18), 7.53 (1H, d, *J* = 2.1 Hz, H14), 7.12 (1H, d, *J* = 8.7 Hz, H27), 7.08 (1H, d, *J* = 8.4 Hz, H17), 3.85 (1H, s, H30), 3.84 (1H, s, H32), 3.83 (1H, s, H20), 3.81 (1H, s, H22). ^13^C {^1^H} NMR (DMSO-*d*_6_, 125 MHz) *δ* = 165.72 (C12), 159.66 (C1), 155.74 (C3), 153.12 (C26), 152.48 (C16), 149.33 (C25), 148.89(C15), 142.71 (C10), 139.51 (C7), 129.28 (C6), 127.72 (C5), 126.86 (C13), 122.73 (C23), 122.07 (C28), 121.81 (C18), 118.13 (C8), 117.31 (C9), 111.54 (C14), 111.51 (C17), 110.44 (C24), 56.29 (C30), 56.25 (C32), 56.18 (C20), 56.12 (C22).^15^N {^1^H} NMR (DMSO-*d*_6_, 50.69 MHz) *δ* = −211.6591 (N11) (Note: See the Figure 7 above for atom numbering). MS (FAB, m/z): 463 (M^+^ + 1, 100%). Elemental analysis: experimental C, 65.41%; H, 5.02%; N, 5.76%; calculated C, 64.93%: H, 4.80%; N, 6.06%.

#### 4.2.3. *N*, *N*′-(2-((4-(Trifluoromethyl)benzyl)carbamoyl)-1,4-phenylene)bis(3,4-dimethoxybenzamide) (**1b**)

**Figure 8:**
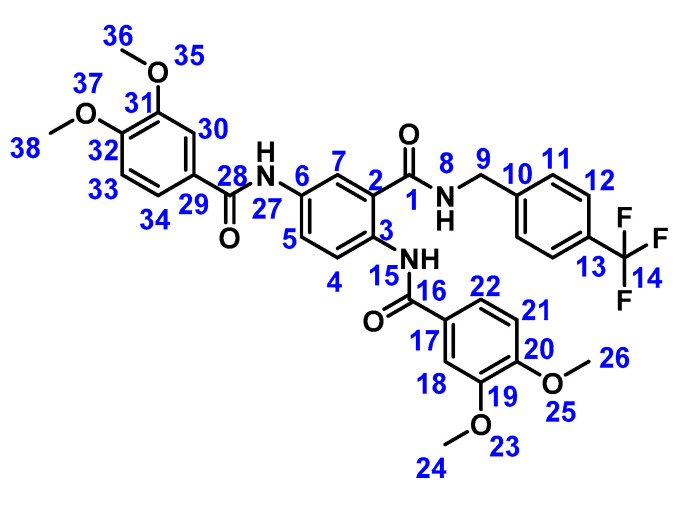
Structure of compound **1b** and atom numbering used for NMR characterization.

Compound **5** (2 g, 4.3 mmol) was added to an Erlenmeyer flask with a magnetic stirrer and an ice bath. Afterwards, 50 mL of DMF were added to the container, and the reaction mixture was stirred for 20 min at 5 °C until the precipitate was completely solubilized. Afterward, 4-(trifluoromethyl) benzylamine (1 g, 5.7 mmol) and 1 mL of acetic acid were added to the solution, which was then vigorously stirred for one day. After reaction completion, 100 mL of ice water were added, and a white precipitate was immediately formed. Finally, 5 mL of a 10% HCl solution were added to the reaction mixture in order to remove the excess of the benzylamine. The precipitate was then filtered, washed with hot hexane, and dried under reduced pressure to give **1a** as a white solid in 72% yield. m.*p*. > 250 °C, IR (KBr, cm^−1^): 3335 (N-H), 2888 (9CH_3_, CH_2_), 1655, 1628 (C=O, amide), 1575 (C=C, aromatic), 1231 (O-CH_3_) ^1^H NMR (DMSO-*d*_6_, 500 MHz) *δ* = 11.82 (1H, s, H15), 10.20 (1H, s, H27), 9.43 (1H, t, *J* = 5.9 Hz, H8), 8.45 (1H, d, *J* = 8.9 Hz, H4), 8.21 (1H, d, *J* = 2.2 Hz, H7), 7.80 (1H, dd, *J* = 2.2, 8.9 Hz, H5), 7.64 (2H, d, *J* = 8.3 Hz, H12), 7.61 (1H, d, *J* = 1.2, 8.3 Hz, H34), 7.53 (3H, m, H11, H30), 7.42 (2H, m, H18, H22), 7.069 (1H, d, *J* = 9.0 Hz, H21), 7.063 (1H, d, *J* = 8.3 Hz, H33), 4.54 (1H, d, *J* = 5.9 Hz, H9), 3.813 (3H, s, H38), 3.810 (3H, s, H36), 3.80 (3H, s, H26), 3.76 (3H, s, H24). ^13^C {^1^H} NMR (DMSO-*d*_6_, 125 MHz) *δ* = 169.30 (C1), 165.33 (C16), 164.47 (C28), 152.38 (C20), 152.25 (C32), 149.14 (C19), 148.85 (C31), 144.44 (C10), 135.30 (C3), 134.74 (C6), 128.54 (11), 127.31 (C17), 127.23 (13C ^2^*J*_C,F_ = 31.0 Hz), 127.03 (C29), 125.45 (C12, ^3^*J*_C,F_ = 2.9 Hz), 125.24 (C5), 125.23 (C14, ^1^*J*_C,F_ = 252.3 Hz), 122.20 (C2), 121.54 (C4, C34), 121.31 (C7), 120.42 (C17), 111.82 (C33), 111.47 (C21), 111.40 (C30), 110.86 (C22), 56.21 (C38, C36), 56.16 (C26), 55.94 (C24), 43.01 (C9).^19^F {^1^H} NMR (DMSO-*d*_6_, 470.62 MHz) *δ* = 5.91. (Note: see Figure 8 above for atom numbering). MS (FAB, m/z): 638 (M^+^ + 1, 100%). Elemental analysis: experimental C, 62.59%; H, 5.01 %; N, 6.28%; calculated C, 62.16%; H, 4.74%; N, 6.59%

### 4.3. In Vivo Evaluation in Metabolic Syndrome

#### 4.3.1. Animals

Forty male Sprague-Dawley male rats with a bodyweight of 250 ± 25 g were housed in acrylic boxes under standard environmental conditions (five animals per cage) with free access to water and food (Purina, Minnetonka, MN) and kept in a clear air room maintained on an artificial 12 h light/dark cycle (lights on at 08:00 h). Animals were treated following the guidelines and requirements of the World Medical Association Declaration of Helsinki and the recommendations of the Official Mexican Standard for the Production, Care and Use of Laboratory Animals (SAGARPA, NOM-062-ZOO-1999). The protocol has the approval of the Institutional Subcommittee for the Care and Use of Laboratory Animals (SICUAL) with registration FMM/SICUAL/006/2017 (approved on 30 August 2017).

#### 4.3.2. MetS Induction and Treatment with Compound **1b**

Animals were randomly assigned to either continue consuming regular Chow commercial food (control groups) (*n* = 20) (Purina-Rodent Laboratory Chow-5001 3.310 kcal/g) or fed with a high-fructose and high-fat diet (HFHF diet groups (4.161 kcal/g)) for 12 weeks (*n* = 20), to induce MetS. The weight, glucose levels, total cholesterol, and triglycerides of each animal were recorded at the initiation of the study and every week after until the completion of each feeding time, using an electronic weighing scale. Systolic blood pressure and heart rate were measured by a non-invasive method using an occlusion tail-cuff in the rat’s tail (Ugo Basile, Biological Research Apparatus, 21025, Italy). Subsequently, in weeks 9 and 12, glucose levels, total cholesterol, and triglycerides in caudal vein blood were measured in fasting conditions of 12 hours, using a rapid monitor of metabolites Accutrend plus (Roche). Due to the previous experience of our research group, it is standardized that MetS is well established at week 12 [41]. After the 12 weeks of induction, control and HFHF groups were redistributed into four new groups: (1) control (CM, *n* = 10); (2) control + **1b** (CM/D, *n* = 10); (3) high fructose and high-fat diet (HFHF-M, *n* = 10), and (4) HFHF + **1b** (HFHF-M/D, *n* = 10). The treatment period lasted 14 days, and the animals were maintained with the same diet they had during the previous 12 weeks. All the animals received 100 μL of a 2% Kolliphor EL mixture in water with or without compound **1b** (10 mg/Kg) via nasogastric. Animals were sacrificed by decapitation. The blood was collected in a test tube and centrifuged at 1372× *g* for 15 minutes at 4 °C. The serum was collected and stored at −80 °C until the completion of the analysis.

#### 4.3.3. In Vivo Acute Toxicity Determination

Experiments were performed on male mice ICR (body weight range, 25–30 g. All experiments were carried out according to the Mexican Official Norm for Animal Care and Handing (NOM-062-ZOO-1999). The experimentation animals were kept with a 12 h light/dark cycle in a climate and light-controlled room. Twelve hours before experiments, food was suspended, but animals kept drinking water ad libitum. Compound **1b** was administrated as an emulsion in Kolliphor EL-water (2%). The concentrations were adjusted with water to administrate 200 µL per 10 g of body weight orally. Mice were treated in two phases. In the first phase, to three groups of three animals each, doses of 10, 100, and 1000 mg/kg of **1b** were administered. In the second phase, the doses were administered according to the Lorke method [68], to two different groups of three animals, each at doses of 1500 mg/kg and 2500 mg/kg. In both phases, mice were observed for signs of toxicity, mortality, or changes in behavioral patterns. At the end of the experiments, the animals were sacrificed in a CO_2_ chamber.

#### 4.3.4. Triacylglycerides, Cholesterol and Glucose Analysis

The animals’ serum was defrosted, and both triacylglycerides cholesterol and glucose were determined through spectrophotometric assays GPO (Teco Diagnostics, Anaheim, CA, USA) following the manufacturer’s instructions. The concentration was calculated using the formula SC = (SA/StdA) × SdtC, where SC: sample concentration expressed in mg/dL, Am: sample absorbance, StdA: standard absorbance, and SdtC: concentration of the standard (200 mg/dL). Statistical analysis was performed using one-way ANOVA, followed by Dunnett’s multiple comparisons test employing GraphPad Prism version 7.04 (GraphPad Software, La Jolla, CA, USA). Differences were considered significant when *p* ≤ 0.05.

## 5. Conclusions

To conclude, suitable models for evaluating the multitarget effects of a drug in a preclinical evaluation are needed to assess the tested compounds’ behavior in a context closer to a clinical situation. The use of the diet-induced MetS model allowed us to simultaneously evaluate the effects of compound **1b** on ameliorating glucose, triacylglyceride, and total cholesterol levels as well as blood pressure. The in-silico evaluation and the in vivo experimentation results suggest that the favorable effects on those parameters are probably attributable to the interaction of **1b** with several targets related to MetS, including PPAR receptors and enzymes like ACE and HMG-CoA reductase. The experimental demonstration of these potential molecular mechanisms of action is currently ongoing.

## Figures and Tables

**Table 1:** Docking scores of the ligands with higher theoretical affinity against some targets related to MetS ^1^.

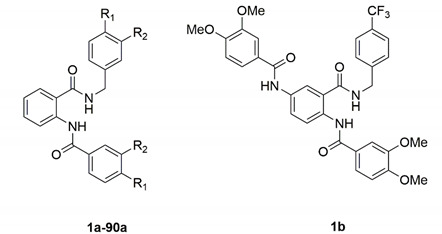						
Ligand.	Benzylamine Substituents	Benzamide Substituents	PPAR-α	PPAR-γ	HMG-CoA Reductase	ACE
**48a**	4′-CF_3_	3′,4′-diOMe	−118.7	−127.9	−111.8	−111.3
**45a**	2′,4′-di-OMe	3′,4′-diOMe	−119.0	−124.2	−113.3	−97.3
**44a**	3′,4′-diOMe	3′,4′-diOMe	−125.0	−119.5	−112.5	−90.9
**28a**	4′-CF_3_	4′-NO_2_	−113.8	−116.7	−99.0	−119.7
**88a**	4′-CF_3_	4′-SO_2_NH_2_	−118.7	−127.9	−111.8	−111.6
**1b**	--	--	−151.6	−165.2	−156.2	−137.2
**Reference ^2^**	--	--	−113.8	−116.7	−164.5	−123.0

^1^ More negative docking scores indicate higher theoretical affinity. ^2^ Reference ligands: AZ 242 (dual PPAR α/γ agonist), atorvastatin (HMG-CoA reductase), lisinopril (ACE).

## Data Availability

Data is contained within the article and Supplementary Material.

## Supplementary Materials

The following are available online at https://www.mdpi.com/article/10.3390/ph14090914/s1, Table S1: complete in silico data, Table S2: Metabolic parameters values mean ± SD), NMR spectra of compounds **1b** and **5**.

## Abbreviations

ACAT	Acyl-coenzyme A cholesterol acyltransferase
ACE	Angiotensin-converting enzyme
ADME/Tox profile	Absorption, Distribution, Metabolism, Excretion and Toxicity profile
CETP	Cholesterylester transfer protein
DPP4	Dipeptidyl peptidase 4
FFAR–1	Free fatty acid receptor -1
FXR	Farnesoid X receptor
hERG	Human ether-à-go-go-related gene
HFHF diet	High fructose and high-fat diet
HMG–CoA reductase	Hydroxymethylglutaryl coenzyme A reductase
MetS	Metabolic syndrome
PPAR	Peroxisome proliferator–activated receptor
PTP1B	Protein tyrosine phosphatase 1B
