# Supplementary material for: In Silico-Based Design and In Vivo Evaluation of an Anthranilic Acid Derivative as a Multitarget Drug in a Diet-Induced Metabolic Syndrome Model
Source: Pharmaceuticals (Basel). 2021 Sep 10;14(9):914. doi: 10.3390/ph14090914 (PMC8466046; doi:10.3390/ph14090914)
Supplement: Supplementary file 1 [file pharmaceuticals-14-00914-s001.zip › pharmaceuticals-1357341-supplementary.pdf]

**Supplementary information for Design and evaluation of an anthranilic acid derivative as a multitarget drug in a diet-induced metabolic syndrome model by González-Álvarez, et.al.**

## 1. Complete docking results

| | | | Dyslipidemia | | | | | | Hyperglycemia | | | | | Hypertension | |
|---|---|---|---|---|---|---|---|---|---|---|---|---|---|---|---|
| Compound | Benzylamine | Benzamide | PPAR-alfa | HMG-CoA reductase | ACAT | CETP | Squalene synthase | FXR | PPAR-gamma | DPP-IV | Aldose reductase | GPR40 | PTP1B | Renin | ACE |
| 1b | 4'-CF3 | 3',4'-diOMe | -151.6 | -156.2 | -121.6 | -129.2 | -118.7 | -132.3 | -165.2 | -84.2 | -136.0 | -91.3 | -107.9 | -104.3 | -137.2 |
| Reference ligands | | | -113.8 | -164.5 | -110.8 | -100.1 | -111.6 | -144.4 | -116.7 | -144.5 | -97.1 | -122.7 | -150.9 | -166.8 | -123.0 |
| 1a | H | 4'-Et | -97.6 | -107.9 | -90.6 | -90.1 | -93.7 | -107.3 | -108.0 | -77.2 | -117.9 | -87.9 | -94.2 | -101.6 | -95.7 |
| 2a | 4'-Me | 4'-Et | -105.8 | -112.9 | -91.1 | -76.9 | -96.3 | -101.8 | -104.0 | -63.2 | -121.5 | -98.8 | -79.0 | -94.5 | -108.3 |
| 3a | 4'-OMe | 4'-Et | -104.6 | -110.0 | -90.0 | -86.9 | -94.6 | -100.0 | -110.6 | -68.6 | -101.8 | -94.6 | -92.0 | -96.3 | -79.2 |
| 4a | 3',4'-diOMe | 4'-Et | -107.9 | -108.0 | -91.5 | -86.7 | -90.6 | -100.3 | -106.5 | -69.3 | -140.0 | -111.7 | -89.8 | -94.4 | -93.4 |
| 5a | 2',4'-diOMe | 4'-Et | -109.1 | -106.5 | -95.2 | -90.1 | -99.6 | -97.9 | -109.7 | -77.4 | -127.3 | -100.9 | -89.4 | -102.8 | -92.7 |
| 6a | 4'-Cl | 4'-Et | -102.9 | -101.8 | -88.5 | -79.8 | -86.0 | -97.5 | -112.2 | -69.5 | -119.1 | -90.5 | -94.5 | -99.3 | -89.2 |
| 7a | 4'-pyr | 4'-Et | -105.8 | -123.3 | -89.6 | -89.3 | -98.4 | -107.8 | -114.7 | -67.8 | -106.8 | -88.5 | -104.5 | -103.1 | -98.5 |
| 8a | 4'-CF3 | 4'-Et | -109.7 | -108.1 | -96.1 | -87.9 | -94.4 | -104.5 | -112.1 | -83.3 | -109.5 | -108.5 | -93.4 | -102.3 | -104.3 |
| 9a | 4'-COOH | 4'-Et | -91.7 | -91.4 | -78.1 | -66.3 | -80.9 | -88.0 | -93.3 | -49.3 | -106.6 | -84.1 | -75.0 | -99.6 | -78.1 |
| 10a | 4'-NMe2 | 4'-Et | -104.0 | -103.3 | -89.3 | -83.2 | -99.9 | -97.3 | -104.6 | -80.5 | -124.4 | -99.9 | -90.9 | -89.2 | -106.8 |
| 11a | H | 4'-OMe | -101.4 | -105.1 | -90.0 | -80.8 | -88.2 | -100.7 | -103.4 | -72.1 | -125.4 | -95.3 | -95.1 | -94.2 | -104.5 |
| 12a | 4'-Me | 4'-OMe | -105.8 | -107.5 | -84.3 | -86.0 | -92.6 | -94.4 | -113.7 | -60.6 | -115.3 | -85.6 | -78.6 | -90.2 | -97.3 |
| 13a | 4'-OMe | 4'-OMe | -99.3 | -102.6 | -90.5 | -88.8 | -87.4 | -101.4 | -101.4 | -69.9 | -131.5 | -92.2 | -86.8 | -100.1 | -97.7 |
| 14a | 3',4'-diOMe | 4'-OMe | -112.8 | -106.3 | -89.3 | -80.3 | -96.7 | -95.0 | -110.4 | -62.3 | -121.9 | -95.1 | -88.9 | -100.5 | -105.4 |
| 15a | 2',4'-di-OMe | 4'-OMe | -115.8 | -106.7 | -92.7 | -84.6 | -100.6 | -92.6 | -112.8 | -61.3 | -126.8 | -99.1 | -91.9 | -103.2 | -97.3 |
| 16a | 4'-Cl | 4'-OMe | -104.8 | -104.3 | -91.9 | -82.5 | -86.8 | -102.2 | -105.9 | -66.8 | -123.1 | -95.1 | -99.3 | -95.4 | -100.0 |
| 17a | 4'-CF3 | 4'-OMe | -101.1 | -106.8 | -95.5 | -84.3 | -89.5 | -99.2 | -112.5 | -61.5 | -122.3 | -95.6 | -93.4 | -93.5 | -104.2 |
| 17a | 4'-CF3 | 4'-OMe | -103.9 | -101.5 | -94.4 | -89.1 | -88.2 | -98.0 | -114.3 | -61.3 | -119.9 | -98.4 | -94.2 | -95.5 | -105.2 |
| 19a | 4'-COOH | 4'-OMe | -106.6 | -109.2 | -95.9 | -82.7 | -87.0 | -102.3 | -109.6 | -58.3 | -123.3 | -100.2 | -90.6 | -93.8 | -80.1 |
| 20a | 4'-NMe2 | 4'-OMe | -111.0 | -108.9 | -96.9 | -73.7 | -91.0 | -89.9 | -104.5 | -69.6 | -126.2 | -99.7 | -91.5 | -90.6 | -99.0 |
| 21a | H | 4'-NO2 | -99.5 | -104.7 | -79.2 | -84.8 | -84.7 | -103.4 | -110.4 | -64.0 | -125.2 | -86.4 | -100.6 | -102.8 | -102.0 |
| 22a | 4'-Me | 4'-NO2 | -105.7 | -97.1 | -93.8 | -75.2 | -81.7 | -95.1 | -116.2 | -64.2 | -127.0 | -89.2 | -104.6 | -99.2 | -105.2 |
| 23a | 4'-OMe | 4'-NO2 | -103.0 | -105.7 | -90.4 | -83.1 | -82.2 | -98.3 | -108.3 | -59.2 | -121.8 | -94.9 | -87.7 | -88.3 | -94.1 |
| 24a | 3',4'-diOMe | 4'-NO2 | -111.4 | -113.9 | -91.5 | -91.1 | -90.0 | -100.3 | -112.8 | -77.2 | -121.2 | -109.3 | -93.4 | -91.6 | -90.8 |

| | | | | | | | | | | | | | | |
|---|---|---|---|---|---|---|---|---|---|---|---|---|---|---|
| 25a | 2',4'-di-OMe | 4'-NO2 | -111.7 | -103.3 | -88.2 | -86.8 | -85.5 | -92.0 | -108.4 | -67.6 | -117.4 | -99.3 | -94.9 | -96.9 | -45.6 |
| 26a | 4'-Cl | 4'-NO2 | -106.1 | -106.4 | -91.6 | -86.6 | -83.3 | -103.7 | -105.8 | -75.0 | -112.6 | -89.4 | -102.1 | -99.4 | -93.9 |
| 27a | 4'-pyr | 4'-NO2 | -101.8 | -103.1 | -92.4 | -87.2 | -88.8 | -94.0 | -106.0 | -73.6 | -123.0 | -87.9 | -98.0 | -99.6 | -99.1 |
| 28a | 4'-CF3 | 4'-NO2 | -113.8 | -99.0 | -90.9 | -84.2 | -87.2 | -99.9 | -116.7 | -64.3 | -121.1 | -109.5 | -106.1 | -98.7 | -119.7 |
| 29a | 4'-COOH | 4'-NO2 | -107.1 | -98.6 | -94.6 | -79.6 | -91.1 | -100.1 | -111.9 | -70.3 | -126.8 | -97.0 | -92.8 | -98.0 | -89.5 |
| 30a | 4'-NMe2 | 4'-NO2 | -107.3 | -116.5 | -100.8 | -81.4 | -92.3 | -99.9 | -107.6 | -58.5 | -116.7 | -104.2 | -93.8 | -92.4 | -104.1 |
| 31a | H | 3',5'-diNO2 | -109.6 | -109.7 | -93.6 | -93.0 | -95.4 | -88.7 | -123.5 | -79.8 | -117.1 | -99.3 | -105.0 | -97.0 | -92.9 |
| 32a | 4'-Me | 3',5'-diNO2 | -108.3 | -102.0 | -101.2 | -86.8 | -93.3 | -97.2 | -115.9 | -78.1 | -120.8 | -95.7 | -76.8 | -102.3 | -93.3 |
| 33a | 4'-OMe | 3',5'-diNO2 | -112.8 | -105.2 | -94.8 | -91.9 | -91.3 | -99.2 | -122.6 | -80.3 | -122.3 | -99.6 | -78.2 | -99.5 | -95.3 |
| 34a | 3',4'-diOMe | 3',5'-diNO2 | -111.4 | -121.0 | -94.8 | -91.5 | -93.3 | -91.9 | -110.1 | -82.2 | -127.2 | -113.0 | -94.4 | -98.3 | -106.2 |
| 35a | 2',4'-diOMe | 3',5'-diNO2 | -114.9 | -114.3 | -96.2 | -88.5 | -92.0 | -98.8 | -118.4 | -84.2 | -115.4 | -101.6 | -100.8 | -110.0 | -90.9 |
| 36a | 4'-Cl | 3',5'-NO2 | -108.4 | -105.0 | -86.7 | -87.4 | -88.7 | -100.5 | -116.9 | -79.9 | -120.7 | -96.1 | -83.4 | -102.3 | -96.4 |
| 37a | 4'-pyr | 3',5'-diNO2 | -108.0 | -109.1 | -91.1 | -94.4 | -93.4 | -89.4 | -114.5 | -84.2 | -119.4 | -92.7 | -102.6 | -102.0 | -91.1 |
| 38a | 4'-CF3 | 3',5'-diNO2 | -113.6 | -98.4 | -102.0 | -90.5 | -98.5 | -87.2 | -122.7 | -88.1 | -142.0 | -107.1 | -86.1 | -99.3 | -99.3 |
| 39a | 4'-COOH | 3',5'-diNO2 | -112.8 | -105.9 | -109.9 | -94.4 | -94.6 | -91.6 | -125.2 | -64.9 | -136.9 | -99.3 | -94.3 | -105.7 | -87.0 |
| 40a | 4'-NMe2 | 3',5'-diNO2 | -107.1 | -106.8 | -110.8 | -84.7 | -88.9 | -97.3 | -114.6 | -62.0 | -127.4 | -99.5 | -100.3 | -108.6 | -83.7 |
| 41a | H | 3',4'-diOMe | -104.8 | -103.0 | -102.8 | -87.3 | -90.2 | -94.3 | -108.9 | -72.8 | -130.9 | -92.8 | -92.7 | -104.9 | -98.1 |
| 42a | 4'-Me | 3',4'-diOMe | -110.6 | -109.4 | -100.4 | -82.0 | -82.0 | -101.3 | -121.4 | -65.6 | -120.5 | -93.9 | -80.7 | -94.7 | -95.9 |
| 43a | 4'-OMe | 3',4'-diOMe | -118.5 | -100.6 | -99.9 | -85.4 | -92.0 | -92.3 | -113.9 | -71.7 | -118.3 | -89.0 | -97.0 | -101.9 | -111.7 |
| 44a | 3',4'-diOMe | 3',4'-diOMe | -125.0 | -112.5 | -100.6 | -95.6 | -99.2 | -103.4 | -119.5 | -72.9 | -132.2 | -95.6 | -104.8 | -101.5 | -90.9 |
| 45a | 2',4'-di-OMe | 3',4'-diOMe | -119.0 | -113.3 | -88.5 | -85.3 | -99.0 | -96.6 | -124.2 | -63.4 | -114.0 | -101.2 | -102.3 | -96.9 | -97.3 |
| 46a | 4'-Cl | 3',4'-diOMe | -105.2 | -106.7 | -96.8 | -91.6 | -94.4 | -106.0 | -115.7 | -68.2 | -123.4 | -90.4 | -98.3 | -101.3 | -108.3 |
| 47a | 4'-pyr | 3',4'-diOMe | -107.3 | -102.6 | -93.3 | -81.7 | -94.4 | -96.2 | -119.2 | -80.9 | -117.3 | -91.8 | -94.5 | -99.4 | -95.8 |
| 48a | 4'-CF3 | 3',4'-diOMe | -118.7 | -111.8 | -91.8 | -88.4 | -100.3 | -118.9 | -127.9 | -72.8 | -140.6 | -90.2 | -105.8 | -100.8 | -98.1 |
| 49a | 4'-COOH | 3',4'-diOMe | -120.1 | -106.6 | -91.0 | -87.0 | -90.8 | -103.3 | -116.8 | -75.9 | -116.4 | -91.0 | -96.9 | -105.8 | -100.5 |
| 50a | 4'-NMe2 | 3',4'-diOMe | -113.9 | -104.7 | -96.3 | -76.3 | -85.5 | -101.9 | -114.7 | -93.7 | -111.7 | -95.0 | -83.9 | -93.0 | -111.3 |
| 51a | H | 4'-Cl | -98.7 | -107.9 | -87.3 | -75.7 | -88.9 | -91.9 | -101.6 | -74.4 | -117.4 | -88.8 | -95.2 | -96.0 | -100.4 |
| 52a | 4'-Me | 4'-Cl | -94.6 | -101.4 | -83.9 | -79.3 | -83.2 | -90.2 | -108.0 | -56.4 | -119.4 | -88.2 | -81.7 | -95.0 | -93.1 |
| 53a | 4'-OMe | 4'-Cl | -99.3 | -108.3 | -83.8 | -79.2 | -86.2 | -100.2 | -108.0 | -70.6 | -111.0 | -97.0 | -90.3 | -93.9 | -86.9 |
| 54a | 3',4'-diOMe | 4'-Cl | -105.7 | -101.7 | -89.4 | -88.3 | -82.6 | -98.4 | -113.0 | -54.2 | -101.4 | -102.6 | -89.6 | -96.9 | -86.8 |
| 55a | 2',4'-diOMe | 4'-Cl | -108.2 | -113.6 | -97.0 | -86.7 | -95.3 | -100.0 | -110.8 | -76.9 | -122.1 | -101.2 | -92.5 | -106.6 | -86.9 |
| 56a | 4'-Cl | 4'-Cl | -101.9 | -105.0 | -91.1 | -75.9 | -80.7 | -94.6 | -108.0 | -76.2 | -113.5 | -90.1 | -84.8 | -92.1 | -99.4 |
| 57a | 4'-pyr | 4'-Cl | -98.4 | -100.8 | -86.4 | -77.0 | -82.0 | -92.0 | -108.6 | -66.6 | -118.9 | -89.1 | -101.7 | -91.7 | -98.2 |

| | | | | | | | | | | | | | | | |
|---|---|---|---|---|---|---|---|---|---|---|---|---|---|---|---|
| 58a | 4'-CF3 | 4'-Cl | -104.9 | -107.9 | -91.4 | -83.8 | -87.2 | -96.6 | -106.8 | -75.3 | -114.7 | -103.1 | -90.2 | -89.6 | -77.6 |
| 59a | 4'-COOH | 4'-Cl | -101.7 | -96.0 | -89.3 | -78.1 | -83.2 | -97.4 | -103.5 | -64.7 | -110.8 | -93.7 | -90.6 | -97.4 | -100.1 |
| 60a | 4'-NMe2 | 4'-Cl | -103.6 | -108.3 | -88.5 | -68.1 | -85.3 | -95.4 | -97.2 | -67.2 | -121.5 | -98.9 | -88.7 | -94.5 | -103.0 |
| 61a | H | 4'-NMe2 | -98.4 | -101.8 | -81.0 | -82.9 | -86.0 | -89.5 | -115.0 | -60.5 | -108.8 | -78.1 | -92.9 | -97.8 | -68.6 |
| 62a | 4'-Me | 4'-NMe2 | -102.6 | -106.4 | -91.5 | -82.2 | -85.5 | -86.5 | -115.6 | -64.2 | -110.7 | -82.6 | -88.6 | -101.6 | -111.1 |
| 63a | 4'-OMe | 4'-NMe2 | -106.9 | -105.1 | -85.9 | -87.0 | -87.2 | -99.8 | -114.1 | -66.3 | -115.2 | -89.0 | -85.7 | -97.9 | -99.1 |
| 64a | 3',4'-diOMe | 4'-NMe2 | -111.6 | -117.2 | -84.3 | -88.9 | -84.8 | -98.9 | -124.3 | -76.3 | -124.2 | -98.5 | -87.0 | -104.5 | -92.6 |
| 65a | 2',4'-diOMe | 4'-NMe2 | -108.3 | -101.7 | -91.4 | -91.1 | -88.1 | -94.2 | -114.4 | -56.7 | -114.2 | -101.4 | -88.8 | -92.2 | -91.0 |
| 66a | 4'-Cl | 4'-NMe2 | -99.0 | -100.7 | -88.9 | -75.1 | -93.9 | -98.4 | -120.3 | -68.1 | -114.3 | -80.0 | -92.2 | -99.8 | -102.9 |
| 67a | 4'-pyr | 4'-NMe2 | -105.6 | -102.5 | -86.9 | -81.0 | -96.8 | -95.7 | -108.0 | -78.9 | -110.8 | -72.8 | -105.3 | -98.4 | -101.0 |
| 68a | 4'-CF3 | 4'-NMe2 | -110.8 | -107.9 | -92.7 | -84.8 | -90.9 | -106.5 | -128.7 | -74.8 | -105.5 | -80.5 | -94.5 | -91.7 | -89.8 |
| 69a | 4'-COOH | 4'-NMe2 | -105.8 | -100.8 | -91.2 | -77.1 | -93.1 | -104.0 | -118.5 | -66.2 | -115.1 | -86.3 | -84.7 | -97.4 | -85.4 |
| 70a | 4'-NMe2 | 4'-NMe2 | -107.3 | -103.7 | -94.8 | -81.2 | -88.7 | -94.1 | -114.6 | -91.1 | -114.1 | -98.0 | -84.4 | -96.0 | -105.1 |
| 71a | H | 4'-CF3 | -104.8 | -103.3 | -90.0 | -91.4 | -82.0 | -101.4 | -112.4 | -74.6 | -122.6 | -90.3 | -100.0 | -98.5 | -65.8 |
| 72a | 4'-Me | 4'-CF3 | -106.5 | -103.9 | -88.4 | -83.6 | -90.8 | -106.8 | -108.0 | -73.4 | -121.4 | -88.4 | -103.2 | -94.4 | -93.2 |
| 73a | 4'-OMe | 4'-CF3 | -105.6 | -108.7 | -88.6 | -87.1 | -90.2 | -99.0 | -104.3 | -61.9 | -103.0 | -98.5 | -85.8 | -94.1 | -104.8 |
| 74a | 3',4'-diOMe | 4'-CF3 | -118.6 | -110.7 | -85.8 | -83.7 | -95.5 | -110.2 | -118.4 | -66.7 | -132.1 | -104.8 | -94.7 | -102.3 | -94.7 |
| 75a | 2',4'-diOMe | 4'-CF3 | -106.9 | -106.4 | -92.1 | -95.3 | -100.3 | -96.8 | -117.5 | -70.5 | -105.9 | -106.9 | -104.1 | -103.8 | -95.0 |
| 76a | 4'-Cl | 4'-CF3 | -107.9 | -110.4 | -94.6 | -81.0 | -93.1 | -109.1 | -114.9 | -70.9 | -123.6 | -92.7 | -99.4 | -99.4 | -102.2 |
| 77a | 4'-pyr | 4-CF3 | -100.1 | -108.0 | -92.0 | -86.0 | -86.8 | -96.6 | -115.0 | -66.2 | -116.9 | -89.2 | -106.5 | -101.1 | -107.3 |
| 78a | 4'-CF3 | 4'-CF3 | -112.8 | -109.5 | -88.0 | -82.4 | -86.7 | -99.7 | -116.8 | -85.0 | -118.4 | -97.8 | -93.5 | -106.9 | -110.8 |
| 79a | 4'-COOH | 4'-CF3 | -112.0 | -106.3 | -83.3 | -83.8 | -89.7 | -105.5 | -116.5 | -85.5 | -117.1 | -103.7 | -90.2 | -93.9 | -89.2 |
| 80a | 4'-NMe2 | 4'-CF3 | -110.1 | -102.7 | -90.3 | -78.1 | -84.2 | -103.6 | -109.5 | -78.9 | -109.6 | -104.0 | -85.7 | -99.2 | -99.8 |
| 81a | H | 4'-SO2NH2 | -108.3 | -112.1 | -90.2 | -90.7 | -92.3 | -107.8 | -111.2 | -72.7 | -112.2 | -87.3 | -110.4 | -99.1 | -103.9 |
| 82a | 4'-Me | 4'-SO2NH2 | -107.9 | -102.1 | -90.0 | -84.7 | -83.0 | -101.1 | -115.6 | -69.1 | -113.1 | -95.7 | -114.4 | -92.7 | -117.8 |
| 83a | 4'-OMe | 4'-SO2NH2 | -108.8 | -101.3 | -95.5 | -88.7 | -91.0 | -102.8 | -115.8 | -72.6 | -115.7 | -98.2 | -88.6 | -84.3 | -89.1 |
| 84a | 3',4'-diOMe | 4'-SO2NH2 | -116.5 | -105.5 | -100.9 | -93.8 | -100.5 | -100.6 | -119.7 | -85.2 | -111.2 | -108.7 | -103.9 | -104.7 | -93.1 |
| 85a | 2',4'-di-Ome | 4'-SO2NH2 | -114.3 | -113.3 | -102.3 | -90.2 | -98.2 | -111.9 | -123.2 | -68.9 | -118.8 | -104.1 | -114.7 | -97.0 | -87.6 |
| 86a | 4'-Cl | 4'-SO2NH2 | -111.4 | -114.9 | -87.9 | -82.0 | -90.9 | -102.8 | -111.5 | -71.1 | -113.3 | -89.2 | -111.3 | -101.8 | -99.7 |
| 87a | 4'-pyr | 4'-SO2NH2 | -106.7 | -101.4 | -92.7 | -87.8 | -85.4 | -97.1 | -112.5 | -73.8 | -113.8 | -87.9 | -112.7 | -101.4 | -103.1 |
| 88a | 4'-CF3 | 4'-SO2NH2 | -112.9 | -103.0 | -102.7 | -90.0 | -88.8 | -99.6 | -119.0 | -80.6 | -109.8 | -88.3 | -118.9 | -102.9 | -111.3 |
| 89a | 4'-COOH | 4'-SO2NH2 | -111.4 | -101.5 | -95.9 | -82.7 | -92.0 | -104.0 | -115.1 | -66.2 | -119.4 | -85.3 | -120.2 | -95.6 | -86.0 |
| 90a | 4'-NMe2 | 4'-SO2NH2 | -108.9 | -108.5 | -91.3 | -77.2 | -84.7 | -98.6 | -122.6 | -72.5 | -111.1 | -106.4 | -93.8 | -96.6 | -102.9 |

**2. NMR spectra**

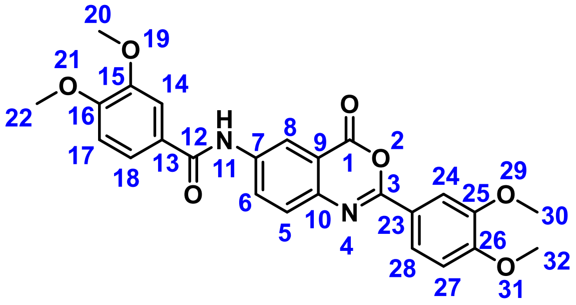


$^{1}H$ NMR (DMSO-$d_6$, 500 MHz) $\delta$ = 10.44 (1H, s, H11), 8.60 (1H, d, $J$ = 2.7 Hz, H8), 8.24 (1H, dd, $J$ = 2.7, 9.1 Hz, H6), 7.75 (1H, dd, $J$ = 1.8, 8.7 Hz, H28), 7.68 (1H, d, $J$ = 8.8 Hz, H5), 7.64 (1H, d, $J$ = 1.8 Hz, H24), 7.62 (1H, dd, $J$ = 2.1, 8.4 Hz, H18), 7.53 (1H, d, $J$ = 2.1 Hz, H14), 7.12 (1H, d, $J$ = 8.7 Hz, H27), 7.08 (1H, d, $J$ = 8.4 Hz, H17), 3.85 (1H, s, H30), 3.84 (1H, s, H32), 3.83 (1H, s, H20), 3.81 (1H, s, H22).

$^{13}C$ {$^{1}H$} NMR (DMSO-$d_6$, 125 MHz) $\delta$ = 165.72 (C12), 159.66 (C1), 155.74 (C3), 153.12 (C26), 152.48 (C16), 149.33 (C25), 148.89(C15), 142.71 (C10), 139.51 (C7), 129.28 (C6), 127.72 (C5), 126.86 (C13), 122.73 (C23), 122.07 (C28), 121.81 (C18), 118.13 (C8), 117.31 (C9), 111.54 (C14), 111.51 (C17), 110.44 (C24), 56.29 (C30), 56.25 (C32), 56.18 (C20), 56.12 (C22).

$^{15}N$ {$^{1}H$} NMR (DMSO-$d_6$, 50.69 MHz) $\delta$ = -211.6591 (N11).

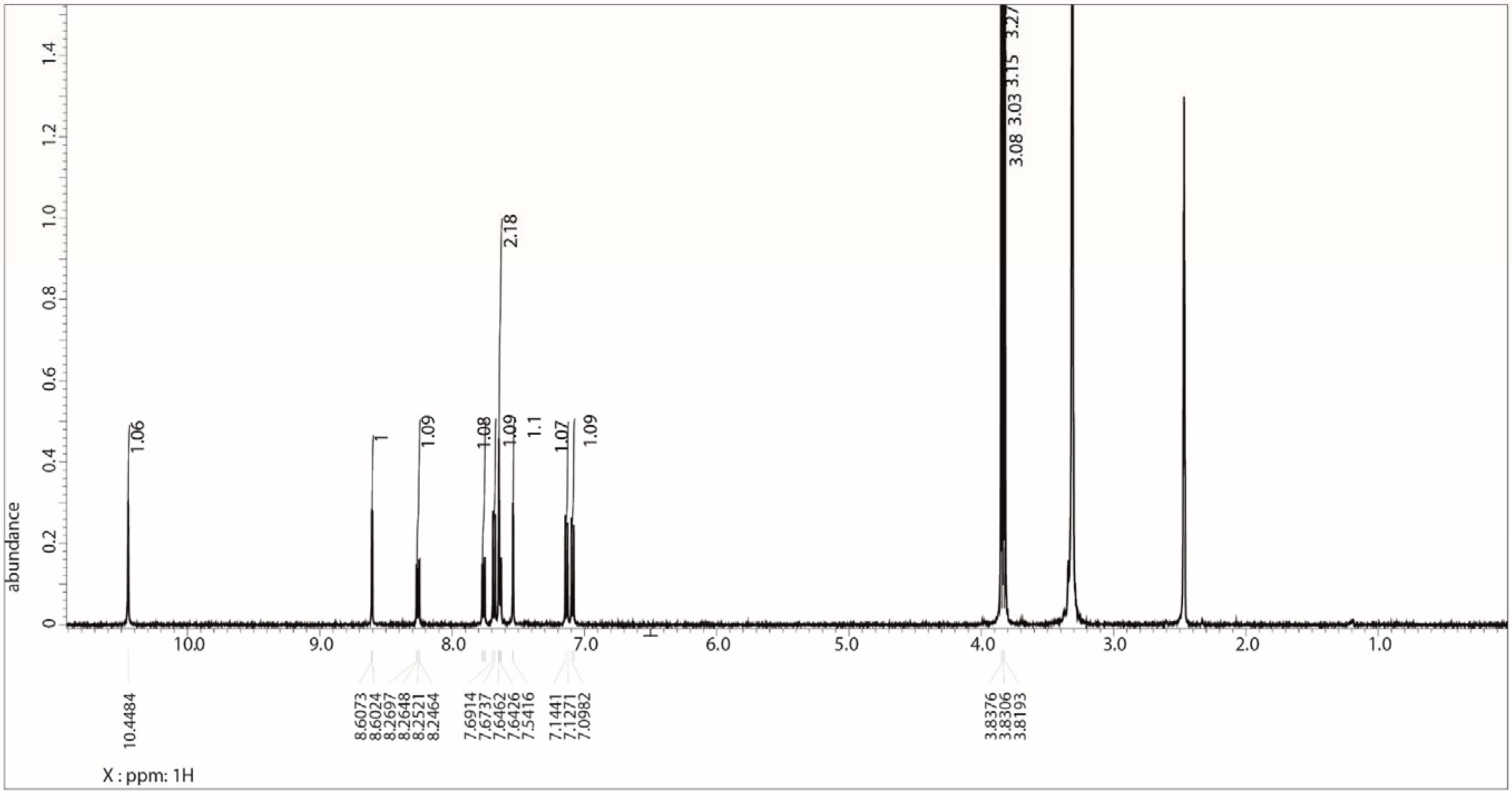
abundance
0
0.2
0.4
0.6
0.8
1.0
1.2
1.4
1.06
1
1.09
1.08
2.18
1.09
1.1
1.07
1.09
3.08 3.03 3.15 3.27
10.0
9.0
8.0
7.0
6.0
5.0
4.0
3.0
2.0
1.0
10.4484
8.6073
8.6024
8.2697
8.2648
8.2521
8.2464
7.6914
7.6737
7.6462
7.6426
7.5416
7.1441
7.1271
7.0982
3.8376
3.8306
3.8193
X : ppm: 1H

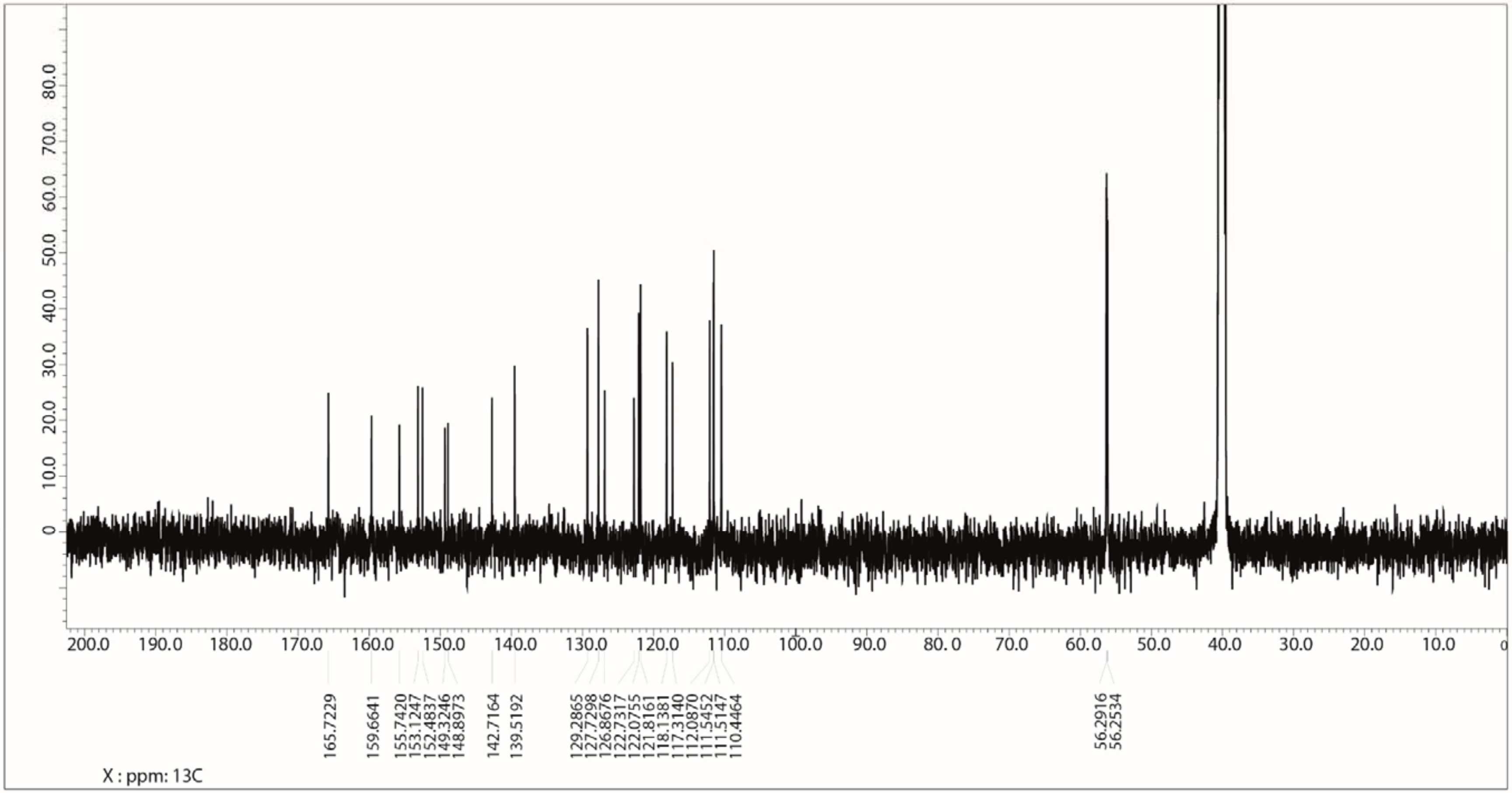
0
10.0
20.0
30.0
40.0
50.0
60.0
70.0
80.0
200.0
190.0
180.0
170.0
160.0
150.0
140.0
130.0
120.0
110.0
100.0
90.0
80.0
70.0
60.0
50.0
40.0
30.0
20.0
10.0
165.7229
159.6641
155.7420
153.1247
152.4837
149.3246
148.8973
142.7164
139.5192
129.2865
127.7298
126.8676
122.7317
122.0755
121.8161
118.1381
117.3140
112.0870
111.5452
111.5147
110.4464
56.2916
56.2534
X : ppm: 13C

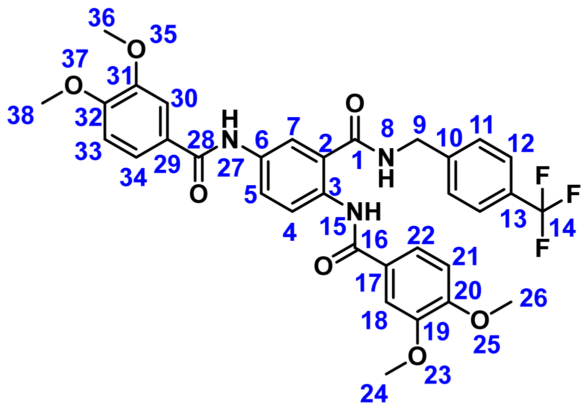


$^{1}$H NMR (DMSO-$d_6$, 500 MHz) $\delta$ = 11.82 (1H, s, H15), 10.20 (1H, s, H27), 9.43 (1H, t, $J$ = 5.9 Hz, H8), 8.45 (1H, d, $J$ = 8.9 Hz, H4), 8.21 (1H, d, $J$ = 2.2 Hz, H7), 7.80 (1H, dd, $J$ = 2.2, 8.9 Hz, H5), 7.64 (2H, d, $J$ = 8.3 Hz, H12), 7.61 (1H, d, $J$ = 1.2, 8.3 Hz, H34), 7.53 (3H, m, H11, H30), 7.42 (2H, m, H18, H22), 7.069 (1H, d, $J$ = 9.0 Hz, H21), 7.063 (1H, d, $J$ = 8.3 Hz, H33), 4.54 (1H, d, $J$ = 5.9 Hz, H9), 3.813 (3H, s, H38), 3.810 (3H, s, H36), 3.80 (3H, s, H26), 3.76 (3H, s, H24).

$^{13}$C {$^{1}$H} NMR (DMSO-$d_6$, 125 MHz) $\delta$ = 169.30 (C1), 165.33 (C16), 164.47 (C28), 152.38 (C20), 152.25 (C32), 149.14 (C19), 148.85 (C31), 144.44 (C10), 135.30 (C3), 134.74 (C6), 128.54 (11), 127.31 (C17), 127.23 (13C $^{2}J_{C,F}$ = 31.0 Hz), 127.03 (C29), 125.45 (C12, $^{3}J_{C,F}$ = 2.9 Hz), 125.24 (C5), 125.23 (C14, $^{1}J_{C,F}$ = 252.3 Hz), 122.20 (C2), 121.54 (C4, C34), 121.31 (C7), 120.42 (C17), 111.82 (C33), 111.47 (C21), 111.40 (C30), 110.86 (C22), 56.21 (C38, C36), 56.16 (C26), 55.94 (C24), 43.01 (C9).

$^{19}$F {$^{1}$H} NMR (DMSO-$d_6$, 470.62 MHz) $\delta$ = 5.91.

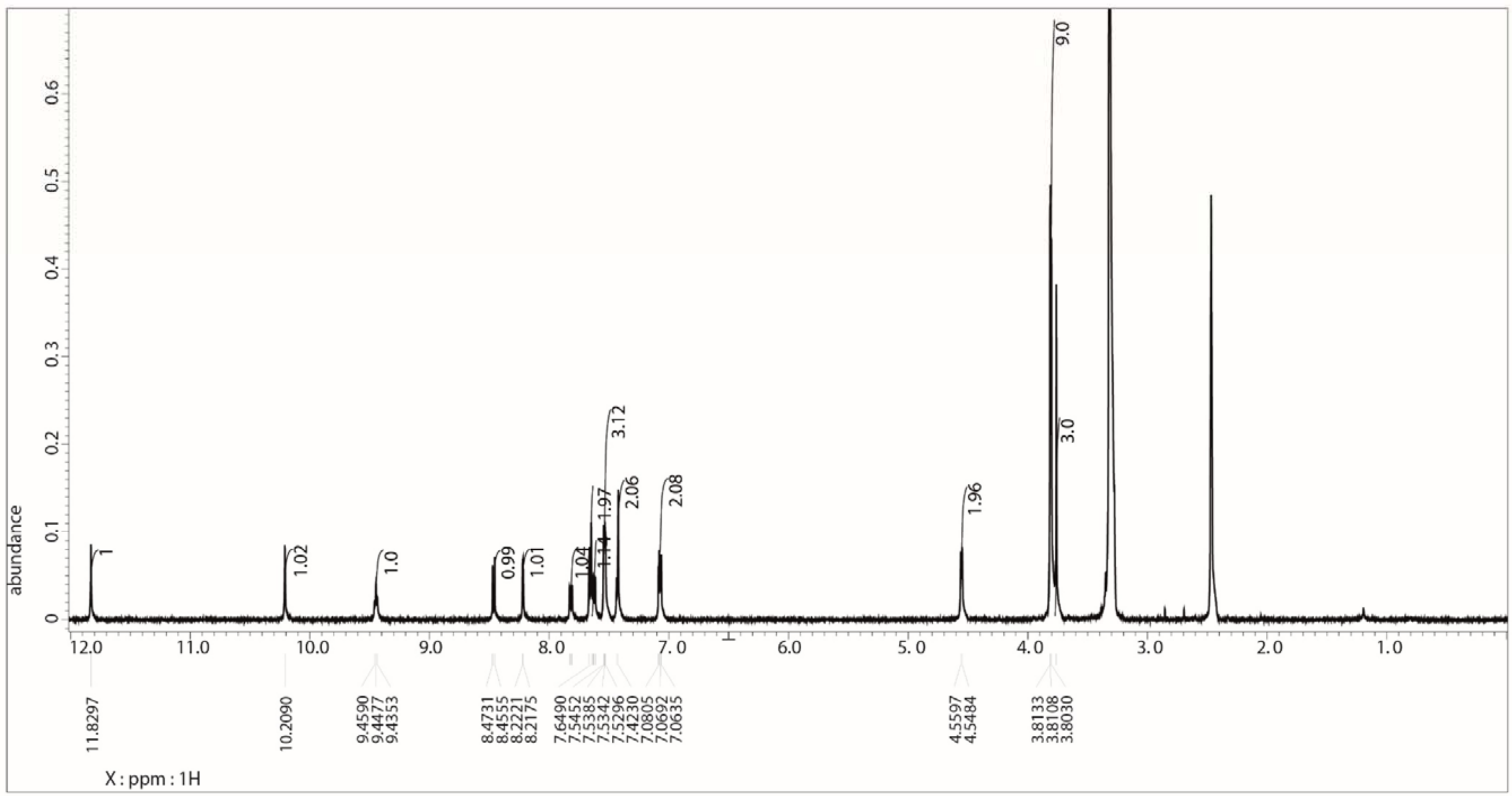

abundance
0
0.1
0.2
0.3
0.4
0.5
0.6
12.0
11.0
10.0
9.0
8.0
7.0
6.0
5.0
4.0
3.0
2.0
1.0
X : ppm : 1H
1
1.02
1.0
0.99
1.01
1.04
1.14
1.97
3.12
2.06
2.08
1.96
9.0
3.0
11.8297
10.2090
9.4590
9.4477
9.4353
8.4731
8.4555
8.2221
8.2175
7.6490
7.5452
7.5385
7.5342
7.5296
7.4230
7.0805
7.0692
7.0635
4.5597
4.5484
3.8133
3.8108
3.8030

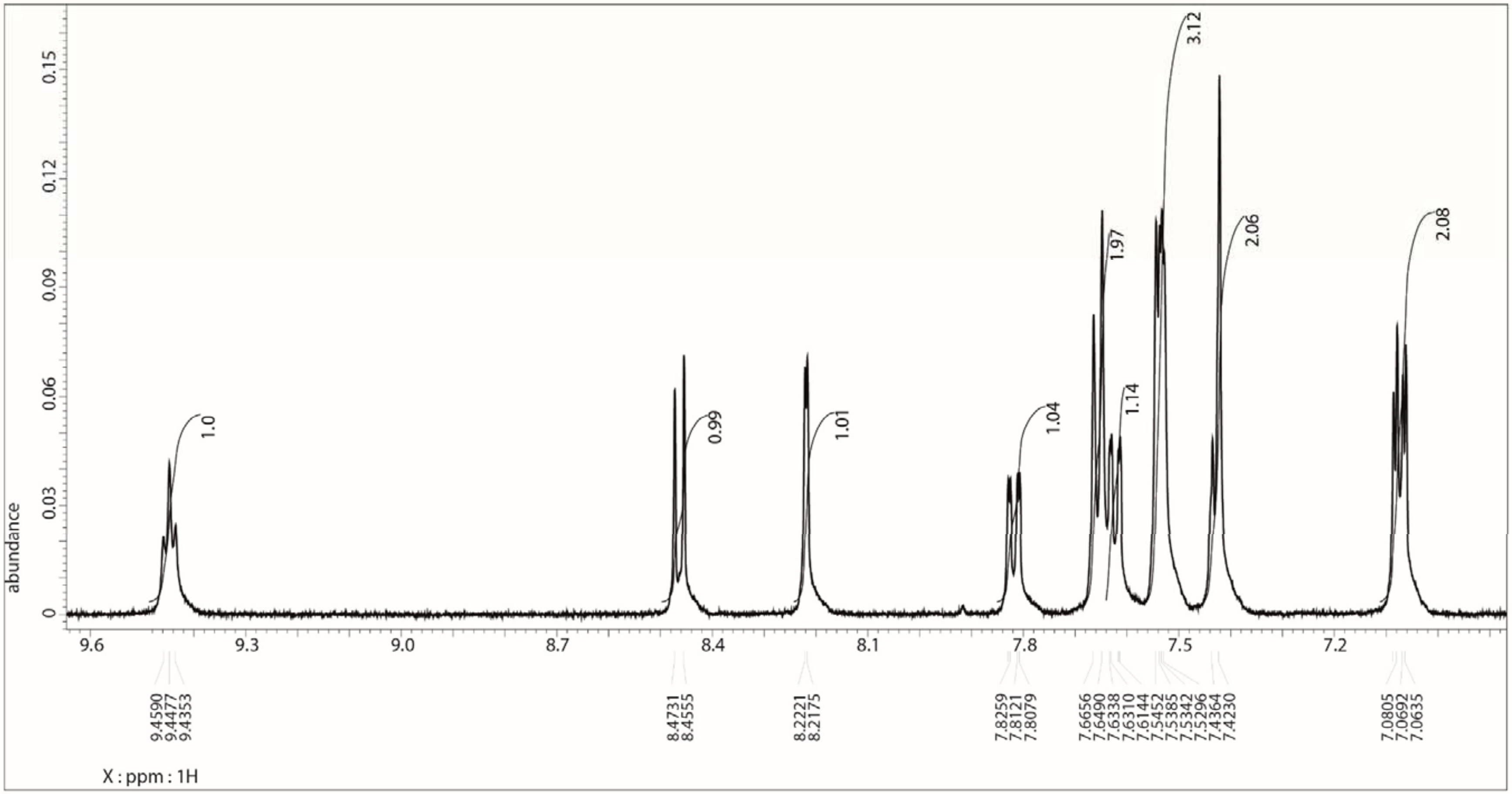

abundance
0.15
0.12
0.09
0.06
0.03
0
1.0
0.99
1.01
1.04
1.97
1.14
3.12
2.06
2.08
9.6
9.3
9.0
8.7
8.4
8.1
7.8
7.5
7.2
9.4590
9.4477
9.4353
8.4731
8.4555
8.2221
8.2175
7.8259
7.8121
7.8079
7.6656
7.6490
7.6338
7.6310
7.6144
7.5452
7.5385
7.5342
7.5296
7.4364
7.4230
7.0805
7.0692
7.0635
X : ppm : 1H

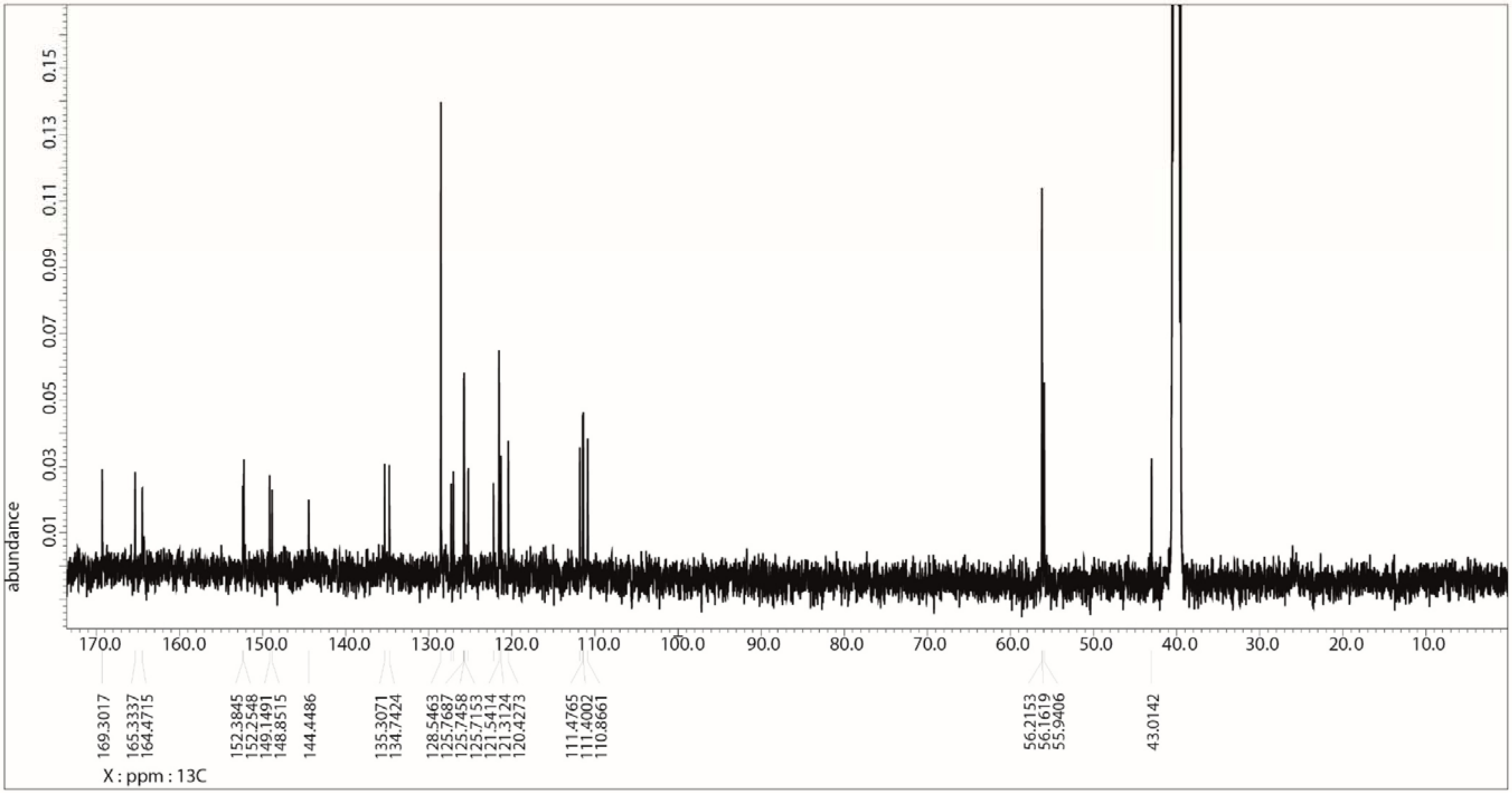

abundance
0.01
0.03
0.05
0.07
0.09
0.11
0.13
0.15
170.0
160.0
150.0
140.0
130.0
120.0
110.0
100.0
90.0
80.0
70.0
60.0
50.0
40.0
30.0
20.0
10.0
169.3017
165.3337
164.4715
152.3845
152.2548
149.1491
148.8515
144.4486
135.3071
134.7424
128.5463
125.7687
125.7458
125.7153
121.5414
121.3124
120.4273
111.4765
111.4002
110.8661
56.2153
56.1619
55.9406
43.0142
X : ppm : 13C

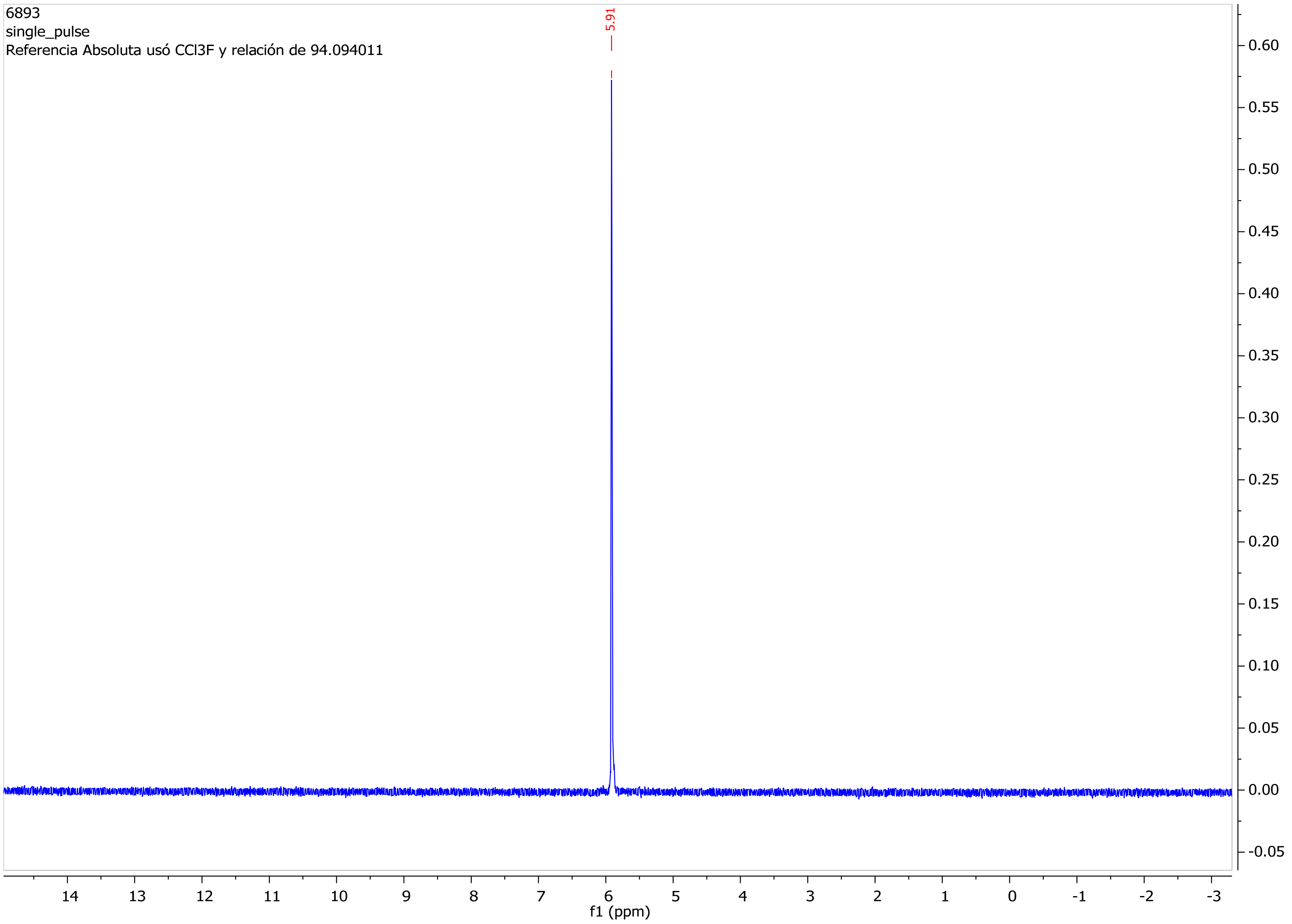

6893
single_pulse
Referencia Absoluta usó CCl3F y relación de 94.094011
5.91
f1 (ppm)

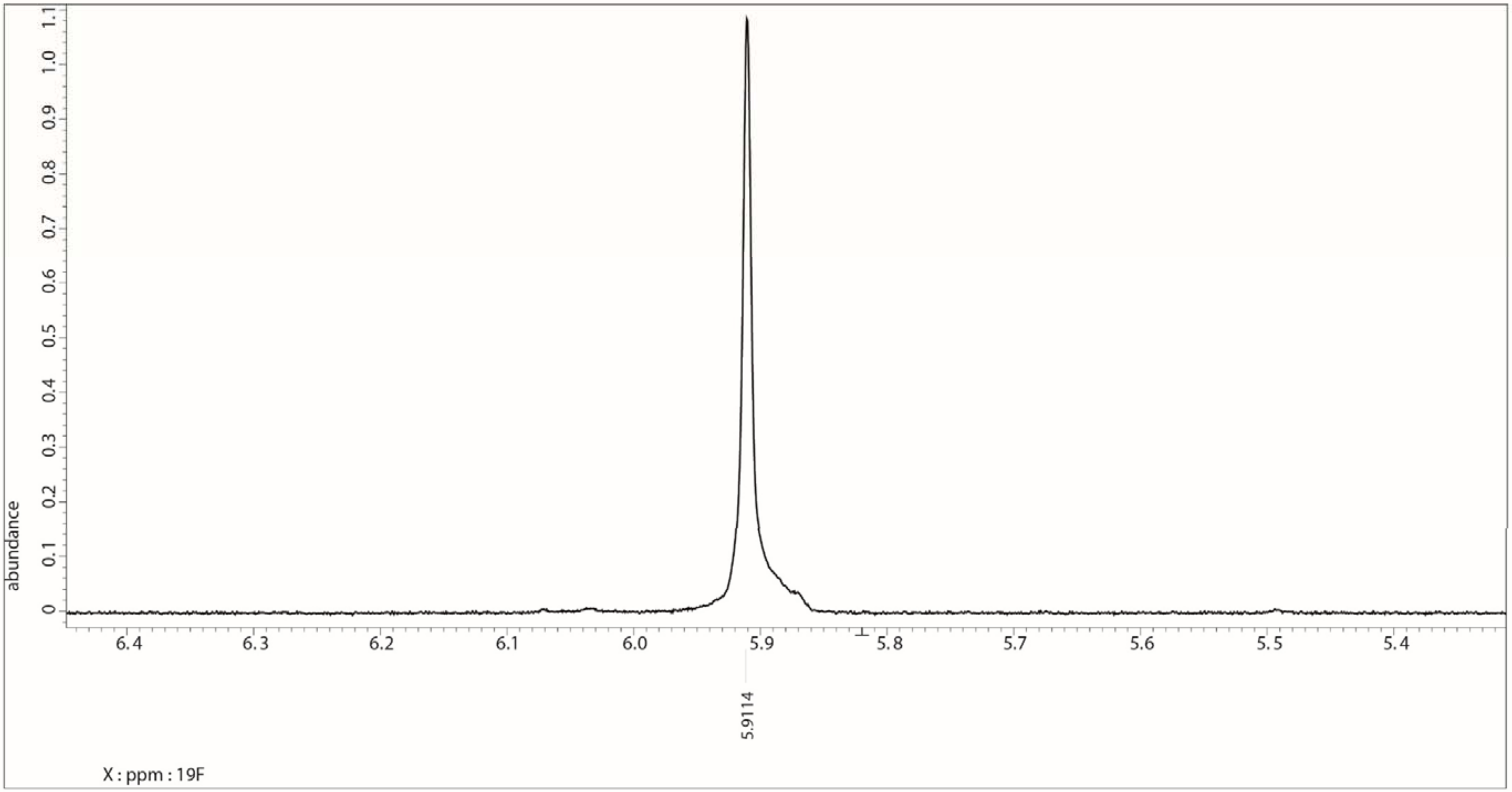
abundance
0
0.1
0.2
0.3
0.4
0.5
0.6
0.7
0.8
0.9
1.0
1.1
6.4
6.3
6.2
6.1
6.0
5.9
5.8
5.7
5.6
5.5
5.4
5.9114
X : ppm : 19F

| Group | METABOLIC SYNDROME PARAMETERS (After 14 weeks) | | | | |
|---|---|---|---|---|---|
| | Weight Change (mean+SD) | Blood Pressure (mean+SD) | Cholesterol (mean+SD) | Glucose (mean+SD) | Triacylglycerides (mean+SD) |
| **CM (n=10)** | 161.0 ± 8.525 | 147.8 ±9.176 | 2.1 ± 0.233 | 6.4 ± 0.686 | 2.1 ± 0.313 |
| **CM/D (n=10)** | 137.9 ± 6.454 | 120.0 ± 9.747 $^{\phi}$ | 2.0 ± 0.202 | 6.7 ± 0.649 | 2.1 ± 0.524 |
| **HFHF (n=10)** | 195.2 ± 15.700 | 197.0 ± 10.002 $^{*}$ | 2.417 ± 0.369 $^{*}$ | 8.0 ± 0.912 $^{*}$ | 3.6 ± 0.715 $^{*}$ |
| **HFHF/D (n=10)** | 178.5 ± 7.959 | 164.3 ± 9.759 $^{*,\$}$ | 1.953 ± 0.140 $^{\$}$ | 7.0 ± 0.588 $^{\$}$ | 2.6 ± 0.676 |

**Table S2.** Effect after 14 days of compound **1b** on metabolic parameters associated to MetS (A) Blood Pressure (B) Cholesterol, (C) Glucose, (D) Triacylglycerides. After 14 days of treatment with compound **1b**, HFHF-M/D group have a statistically significant reduction of blood pressure, total cholesterol and glucose compared to the HFHF-M group. Also, HFHF-M/D group observed a non-statistically reduction of the triacylglycerides compared to the HFHF-M group but also non-statistical significant difference was observed to CM and CM/D groups. Data are presented as means ± Standard Deviation (SD). *Represents statistically significant differences between groups compared to their own control group (CM vs HFHF-M) (CM/D vs HFHF-M/D) ($P < 0.05$). ϕRepresents statistically significant differences between CM vs CM/D ($P < 0.05$). $ Represents statistically significant differences between HFHF-M vs HFHF-M/D ($P < 0.05$).
